# Compositional and temporal division of labor modulates mixed sugar fermentation by an engineered yeast consortium

**DOI:** 10.1038/s41467-024-45011-w

**Published:** 2024-01-26

**Authors:** Jonghyeok Shin, Siqi Liao, Nurzhan Kuanyshev, Yongping Xin, Chanwoo Kim, Ting Lu, Yong-Su Jin

**Affiliations:** 1 Carl R. Woese Institute for Genomic Biology, University of Illinois at Urbana-Champaign, Urbana, IL USA; 2 Synthetic Biology Research Center, Korea Research Institute of Bioscience and Biotechnology (KRIBB), Daejeon, Republic of Korea; 3 Center for Biophysics and Quantitative Biology, University of Illinois at Urbana–Champaign, Urbana, IL USA; 4 Department of Bioengineering, University of Illinois at Urbana–Champaign, Urbana, IL USA; 5 Department of Food Science and Human Nutrition, University of Illinois at Urbana-Champaign, Urbana, IL USA

**Keywords:** Applied microbiology, Metabolic engineering, Computational models, Microbiome

## Abstract

Synthetic microbial communities have emerged as an attractive route for chemical bioprocessing. They are argued to be superior to single strains through microbial division of labor (DOL), but the exact mechanism by which DOL confers advantages remains unclear. Here, we utilize a synthetic *Saccharomyces cerevisiae* consortium along with mathematical modeling to achieve tunable mixed sugar fermentation to overcome the limitations of single-strain fermentation. The consortium involves two strains with each specializing in glucose or xylose utilization for ethanol production. By controlling initial community composition, DOL allows fine tuning of fermentation dynamics and product generation. By altering inoculation delay, DOL provides additional programmability to parallelly regulate fermentation characteristics and product yield. Mathematical models capture observed experimental findings and further offer guidance for subsequent fermentation optimization. This study demonstrates the functional potential of DOL in bioprocessing and provides insight into the rational design of engineered ecosystems for various applications.

## Abstract (web summary)

Synthetic microbial communities are suitable for mixed substrates fermentation and long metabolic pathway engineering. Here, the authors combine fermentation experiments with mathematical modeling to reveal the effect of compositional and temporal changes on division of labor in cellulosic ethanol production using two yeast strains.

## Introduction

Concerns about climate change have accelerated interest in the production of biofuels and chemicals from renewable biomass through microbial fermentation^1,2^. Over the past few decades, metabolic engineering of industrial microorganisms has been increasingly utilized to expand substrate ranges and improve fermentation rates^3,4^. However, excessive genetic perturbations of microbial strains can cause heavy metabolic burdens and high cellular toxicity, resulting in low substrate conversion efficiency and accumulation of undesirable byproducts^5^.

Unlike fermenting microorganisms in industrial conditions, those in nature typically form consortia and divide metabolic processes among participating members to more efficiently utilize complex or otherwise inaccessible substrates, increasing survival in harsh conditions^6,7^. As a result of this DOL, microbial consortia expand the range of substrates that can be degraded, conferring advantages over individual strains^8,9^. Researchers have thus argued that synthetic multi-species communities may outperform individually engineered microorganisms through DOL^10–13^. For example, metabolic pathways for consuming various substrates like glucose, xylose, or arabinose have been separated into multiple strains to build mixed sugar-consuming consortia of synthetic *Escherichia coli*^14,15^ or *Saccharomyces cerevisiae*^16–18^. Longer metabolic pathways have also been divided into multiple functionally specialized strains for high-value chemical production^19–21^.

Although this ecosystem-based strategy has been increasingly explored for bioprocessing, more exact design and implementation of DOL in synthetic microbial communities to optimize desired functions remain underexplored. We argue that alternating the initial community composition and the inoculation times of different strains allows more flexible modulation of the metabolic capacities of individual member strains as well as overall ecosystem behavior, providing tunability of fermentation performance that is difficult for single-strain fermentation. To validate this argument, we combined our fermentation experiments with mathematical modeling to study the effect of compositional and temporal changes on DOL in cellulosic biofuel production from a synthetic consortium.

Using *S. cerevisiae* as our starting microbe, we design and construct an engineered community capable of fermenting glucose and xylose, two sugars prevalent in plant cell wall hydrolysates^22^, as our experimental model system. The DOL in this system is implemented by creating two strains of specialist yeast that exclusively ferment either glucose or xylose in both the absence and presence of the other sugar. Ethanol production from mixed consumption of glucose and xylose is used as the quantifiable output function for the ecosystem. Mathematical models describing the sugar consumption, growth kinetics, and ethanol production of the consortium are then developed to provide quantitative insights as well as predictive guidance. This combined experimental and mathematical study elucidates the functional parameters of DOL towards regulating and optimizing substrate consumption and ethanol production and additionally highlights the strength of ecosystem-based engineering for chemical bioprocessing.

## Results

### Construction of a consortium comprising a glucose and a xylose specialist

We began by engineering a synthetic community that utilizes mixtures of glucose and xylose by using *S. cerevisiae* as a starting strain. *S. cerevisiae* has often been employed to produce biofuels and chemicals from glucose and xylose obtained from lignocellulosic biomass^23,24^. As wild-type *S. cerevisiae* cannot assimilate xylose, metabolic engineering conferring xylose metabolism is necessary^25^. However, this engineered *S. cerevisiae* nonetheless prefers glucose over xylose. Therefore, xylose is only consumed in the absence of glucose^26,27^. Catabolite repression^28,29^ and inhibition of xylose transport by glucose^30,31^ have been identified as the causes of this sequential consumption of glucose over xylose. Although metabolic engineering studies have been undertaken to enable simultaneous consumption of glucose and xylose, the resulting engineered yeast suffer from slow consumption of mixed sugars and inefficient production of target products^31–33^.

**Fig. 1:**
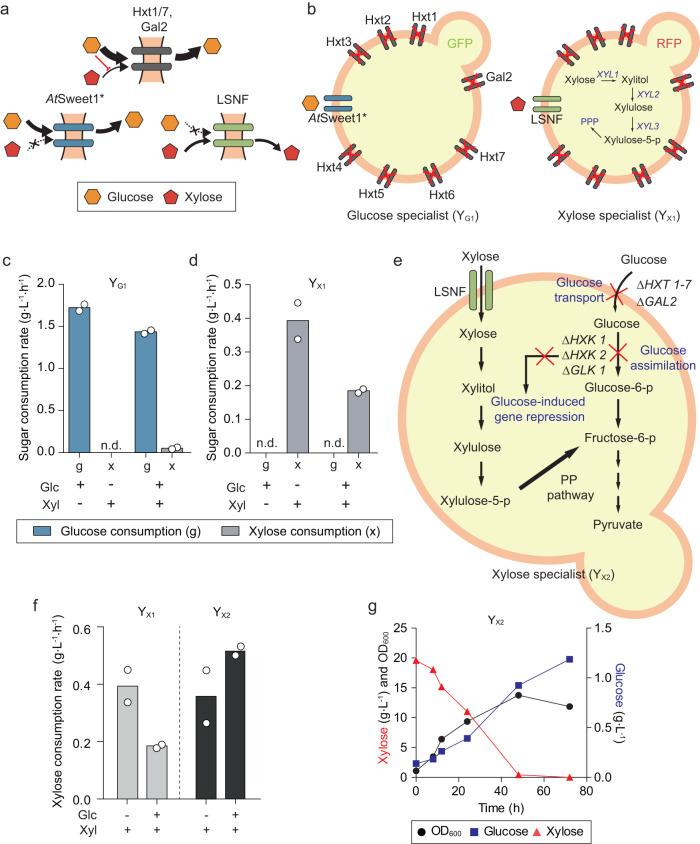
Construction of glucose specialist (Y_G1_) and xylose specialist (Y_X1_). **a** Sugar transporters that transport glucose and/or xylose. Hxt1/7 and Gal2 can transport both glucose and xylose, but glucose transport has priority over xylose. *At*Sweet1* is a transporter that specifically transports glucose, and LSNF is a transporter that specifically transports xylose. **b** Construction of a specific sugar specialist by expressing the specific sugar transporter in SR8D8, a xylose consuming yeast in which the major hexotransporters have been deleted. A glucose specialist (Y_G1_) was constructed by expressing *At*Sweet1*, and a xylose specialist (Y_X1_) was constructed by expressing LSNF, respectively in SR8D8. **c**, **d** Sugar consumption rate under the conditions of glucose only, xylose only, and a mixture of glucose and xylose of the Y_G1_ **c** and Y_X1_ **d**. **e** Alleviation of glucose repression and elimination of glucose consumption pathway in Y_X2_ by deletion of hexokinase and glucokinase. Glucose consumption of the Y_X2_ was inhibited by the deletion of major hexose transporter and hexokinase and glucokinase. **f** The xylose consumption rate of the Y_X1_ and Y_X2_ under the conditions of xylose only or a mixture of glucose and xylose. **g** Glucose production of the Y_X2_ consuming xylose. Results are the mean of replicated experiments (*n* = 2). Source data are provided as a Source Data file.

To overcome this limitation of mixed sugar utilization, we designed DOL in a synthetic microbial community through the creation of two specialist strains that respectively consume glucose and xylose only. Specifically, we used the *S. cerevisiae* SR8D8 strain, which has a xylose assimilation pathway but is deficient in major hexose transporters (Hxt1-7, Gal2), as a parental strain^34^. As the SR8D8 strain cannot transport both glucose and xylose, a glucose-specific transporter and a xylose-specific transporter were introduced into SR8D8 to construct the initial glucose specialist (Y_G_) and xylose specialist (Y_X_) strains. The glucose-specific transporter (*At*Sweet1*) was obtained by laboratory evolution of AtSweet1 from *Arabidopsis thaliana* for improved glucose transport (Fig. 1a and Supplementary Fig. 1). The xylose-specific transporter (LSNF) was obtained by a point mutation (N370F) in a sugar transporter (D2) from *Lipomyces starkeyi*^35^. After introducing green fluorescent protein (GFP) and red fluorescent protein (RFP) expression cassettes for monitoring population changes during co-cultures, we obtained the glucose specialist Y_G1_ and xylose specialist Y_X1_ (Fig. 1b).

Fermentation of single substrates (20 g L^−1^ of glucose or 20 g L^−1^ of xylose alone) showed that Y_G1_ and Y_X1_ are both specialized in consuming their corresponding sugars (Fig. 1c, d). However, when a mixture of glucose and xylose was used, Y_G1_ retained a high rate of consumption relative to the single substrate condition, taking 12 hours to deplete the provided glucose (Fig. 1c and Supplementary Fig. 2c). By contrast, the fermentation rate of Y_X1_ reduced significantly in mixed glucose/xylose media and it took 100 hours after a 24-hour lag period to complete xylose fermentation (Fig. 1d and Supplementary Fig. 2f). These results suggest that glucose delays and inhibits xylose consumption by the Y_X1_ strain.

We reasoned that the hexokinase enzymes and extracellular glucose might be involved in the delay and inhibition of xylose consumption in Y_X1_^36,37^. Therefore, we deleted *HXK1*, *HXK2*, and *GLK1* genes coding for hexokinases and glucokinase in Y_X1_ (Fig. 1e) to create the strain Y_X2_. As expected, Y_X2_ was able to consume xylose rapidly without a lag period, even in the presence of glucose (Fig. 1f and Supplementary Fig. 3d). Interestingly, in Y_X2_, xylose consumption was enhanced in the presence of glucose.

**Fig. 2:**
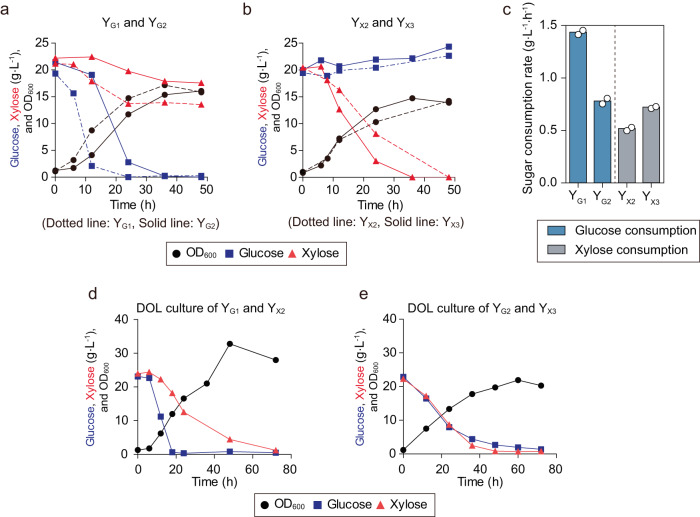
Controlled sugar consumption rates of the Y_G_ and Y_X_. **a** Comparison of glucose consumption between the Y_G1_ and Y_G2_ (Solid line: Y_G2_, dotted line Y_G1_). **b** Comparison of xylose consumption between the Y_X2_ strain and Y_X3_ strain (Solid line: Y_X2_, dotted line Y_X3_). **c** The sugar consumption rate of the Y_G1_, Y_G2_, Y_X2_, and Y_X3_ in the sugar mixture of glucose and xylose. **d**, **e** Glucose and xylose consumption in the co-cultivation of the **d** Y_G1_ and Y_X2_ and the **e** Y_G2_ and Y_X3_. The initial inoculation concentrations of the Y_G1_ and Y_X2_ were mixed at a ratio of 1:1. Results are the mean of replicated experiments (*n* = 2). Source data are provided as a Source Data file.

Despite these improvements, the glucose consumption rate (1.5 g L^−1^ h^−1^) of Y_G1_ was still almost three-fold higher than the xylose consumption rate (0.5 g L^−1^ h^−1^) of Y_X2_ (Fig.1c, f). Because of this consumption rate disparity, the specialist Y_G1_ might dominate in a co-culture and inhibit the growth of Y_X2_ through the production of ethanol, thus resulting in the imbalance growth between the two strains^38^. To match glucose and xylose consumption rates and increase the tunability of the strains, the *At*Sweet* in Y_G1_ was replaced with unevolved *At*Sweet to reduce glucose consumption rate, and an additional copy of the LSNF expression cassette was introduced into Y_X2_ to enhance xylose consumption rate. The resulting strains, Y_G2_ and Y_X3_, exhibited similar glucose and xylose consumption rates (0.8 g L^−1^ h^−1^ and 0.7 g L^−1^ h^−1^), respectively (Fig. 2a–c, Supplementary Fig. 4).

When the original specialists Y_G1_ and Y_X2_ were inoculated at the same cell densities into a mixture of glucose and xylose, glucose was depleted within 24 h, much faster than the more than 72 h needed for xylose (Fig. 2d). In contrast, the optimized specialists, Y_G2_ and Y_X3_, depleted glucose and xylose at the same time when they were inoculated at the same cell density, indicating that this additional engineering successfully allowed simultaneous consumption of glucose and xylose sugars (Fig. 2e). As xylose fermentation often accumulates growth-inhibiting acetate as a byproduct^39^, *ALD6* coding for aldehyde reductase was additionally deleted in Y_G2_ and Y_X3_ to eliminate possible interference of acetate on fermentation progress. The resulting strains were named Y_G2∆*ALD6*_ and Y_X3∆*ALD6*_ (Supplementary Fig. 5).

### Compositional DOL modulates the fermentation performance of the consortium

Enabled by the partitioning of glucose and xylose consumption, our engineered community would in principle allow both separate control and flexible modulation of utilization kinetics for each substrate, influencing the overall production of ethanol through direct alteration of the initial ecosystem composition. This is a unique degree of control that is unavailable for single-engineered strains.

**Fig. 3:**
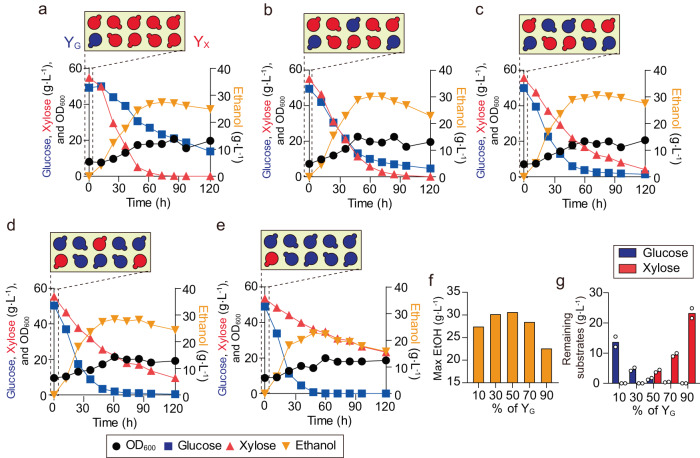
Compositional DOL for the mixed sugar consumption. **a**–**e** Yeast consortium with 1:9 **a**, 3:7 **b**, 5:5 **c**, 7:3 **d**, 9:1 **e** ratio of Y_G_:Y_X_ was treated in YP media containing 50 g L^−1^ of glucose and xylose. In this experiment, Y_G2∆*ALD6*_ and Y_X3∆*ALD6*_ were used as Y_G_ and Y_X_, respectively. Feasibility of simulation in compositional DOL. **f** Maximal ethanol concentration in **a**–**e**. **g** Total remaining glucose and xylose in the culture at hour 120. Results in **a**–**e**, **g** are the mean of replicated experiments (*n* = 2). Source data are provided as a Source Data file.

To test this conceptual reasoning, we performed a set of mixed sugar fermentation experiments with the optimized consortium. We conducted a consortium fermentation of a mixture of 50 g L^−1^ of glucose and 50 g L^−1^ of xylose and modulated initial cell density ratios of the two strains, using ratios of 1:9 (Fig. 3a), 3:7 (Fig. 3b), 1:1 (Fig. 3c), 7:3 (Fig. 3d), and 9:1 (Fig. 3e) of Y_G2∆*ALD6*_ and Y_X3∆*ALD6*_. The results confirmed that, with alteration of initial composition, the synthetic consortium generated distinct sugar consumption dynamics and ethanol production profiles. Notably, by increasing relative initial cell density of Y_G2∆*ALD6*_ in the consortium, the rate of glucose consumption by the ecosystem increased monotonically. At the same time, as the relative initial density of Y_X3∆*ALD6*_ decreased from 1:9 to 9:1, xylose degradation correspondingly reduced monotonically. These results demonstrate effectiveness of initial composition modulation as an approach to control ecosystem fermentation behavior. Additionally, the results showed that, by varying initial composition, both the ethanol production and substrate co-consumption can be systematically optimized (Fig. 3f, g).

### Temporal DOL is an effective strategy to regulate mixed sugar fermentation

**Fig. 4:**
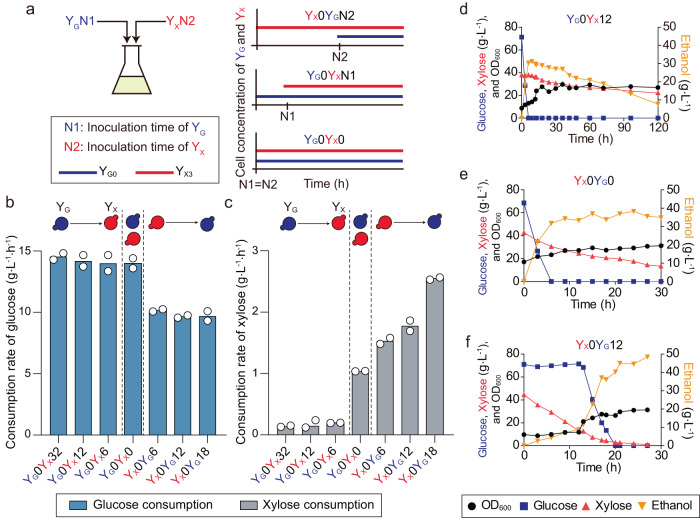
Temporal DOL in mixed sugar consumption. **a** Schematic diagram of temporal DOL to consume mixed sugar. The cell concentrations of Y_G_ and Y_X_ vary with the inoculation time of Y_G_ (N1) and Y_X_ (N2). **b**, **c** Consumption rates of glucose **b** and xylose **c** of the consortium under different temporal inoculation conditions. **d**–**f** Temporal profiles of mixed substrate fermentation under three specific temporal settings: Y_G_0Y_X_12 (d), Y_X_0Y_G_0 **e**, and Y_X_0Y_G_12 **f**. Results are the mean of replicated experiments (*n* = 2). Source data are provided as a Source Data file.

Parallel to compositional division, temporal partition of labor by introducing community members into fermentation at different time points provides an alternative way of DOL. Because ethanol has been shown to inhibit xylose consumption more severely than glucose consumption in engineered yeast^38^, simultaneous co-utilization of glucose and xylose will impair the use of xylose more significantly than the assimilation of glucose due to the accumulation of ethanol. While in theory chemostat fermentation could reduce ethanol toxicity in mixed sugar fermentation, the risk of strain contamination and high product recovery cost could make it difficult to use in practice^40^. Additionally, the glucose specialist might indirectly inhibit xylose consumption by removing the dissolved oxygen that is highly required for xylose consumption^41^. If mixed sugars are used sequentially, with glucose first and then xylose, xylose consumption would be further aggravated due to inhibitory effects by ethanol from glucose fermentation. As such, reversing this sequence with use of xylose before glucose might resolve this severe ethanol sensitivity of xylose fermentation and increase the oxygen availability for xylose consumption. Given the DOL design of our engineered consortium, this reverse order utilization of xylose and glucose is feasible in our ecosystem by inoculating the xylose specialist before the glucose specialist (Fig. 4a).

As such, we conducted experiments using the consortium with different inoculation times for glucose and xylose specialist strains. To maximize ethanol production from glucose in the presence of high concentrations of ethanol produced by the initial xylose fermentation, we first optimized the consumption rate of the glucose specialist by deleting *ALD6* to minimize acetate production in the wild-type *S. cerevisiae* D452-2 while expressing GFP to monitor cell density (Supplementary Fig. 6). The resulting strain Y_G0∆*ALD6*_ exhibited the highest glucose consumption rate among all glucose specialists we constructed (Supplementary Fig. 7). Y_G0∆*ALD6*_ and the xylose-fermenting specialist Y_X3∆*ALD6*_ formed the synthetic ecosystem for subsequent experimentation.

When a mixture of 70 g L^−1^ of glucose and 40 g L^−1^ of xylose was fermented by the synthetic consortium with different inoculation times for Y_G0∆*ALD6*_ and Y_X3∆*ALD6*_, we indeed observed different rates of glucose and xylose consumptions (Fig. 4b, c) as well as differing ethanol production patterns (Supplementary Fig. 8). When glucose fermentation by Y_G0∆*ALD6*_ was initiated before xylose fermentation by Y_X3∆*ALD6*_, rapid fermentation of glucose quickly produced ethanol and xylose fermentation was inhibited (Fig. 4d and Supplementary Fig. 8a, b). However, when xylose fermentation by Y_X3∆*ALD6*_ started before glucose fermentation, glucose consumption by Y_G0∆*ALD6*_ was not inhibited by accumulated ethanol. As a result, overall ethanol productivity increased dramatically (Fig. 4f and Supplementary Fig. 8c, d). Additionally, we noticed that the xylose consumption in xylose-first fermentation under 40 g L^−1^ ethanol was generally faster than that in glucose-first fermentation under even lower ethanol concentration, suggesting factors other than ethanol inhibition, such as oxygen deficiency, may contribute to the slowdown of xylose fermentation. These data show that inhibition of xylose fermentation by ethanol and possible oxygen deficiency can be alleviated through temporal partitioning of glucose and xylose fermentation with xylose being consumed first.

### Mathematical modeling captures the characteristics of compositional and temporal DOL

**Fig. 5:**
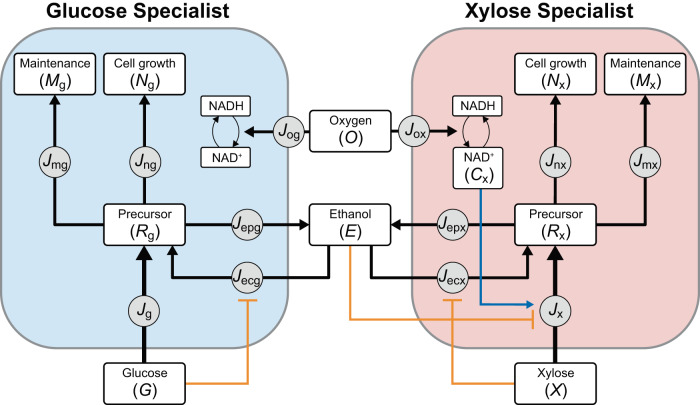
Mathematical modeling of the consortium composed of the xylose and glucose specialists. For each of the specialists, its carbon substrate is consumed to produce a corresponding precursor which is subsequently converted to maintain cell viability, produce biomass and secrete ethanol. Here, the black arrows refer to carbon fluxes, the blunt-ended orange arrows indicate inhibitory regulation from substrates, whereas the blue arrow illustrates the participation of cofactors in substrate utilization.

To quantitatively understand the fermentation results and elucidate the design rules for substrate co-utilization, we developed a mathematical model using an ordinary differential equation-based approach. As shown in Fig. 5, the model considers a glucose specialist and a xylose specialist which consume their corresponding substrates to maintain cellular viability, grow biomass, and produce ethanol as the target product of fermentation. For each specialist, its carbon metabolism centers around the conservation of their respective precursor pool (${R}_{{{{{{\rm{g}}}}}}}$ and ${R}_{{{{{{\rm{x}}}}}}}$), which involves the influxes from sugar consumption (${J}_{{{{{{\rm{g}}}}}}}$ and ${J}_{{{{{{\rm{x}}}}}}}$) and ethanol reassimilation (${J}_{{{{{{\rm{ecg}}}}}}}$ and ${J}_{{{{{{\rm{ecx}}}}}}}$) as well as the outfluxes to cellular maintenance (${J}_{{{{{{\rm{mg}}}}}}}$ and ${J}_{{{{{{\rm{mx}}}}}}}$), cell growth (${J}_{{{{{{\rm{ng}}}}}}}$ and ${J}_{{{{{{\rm{nx}}}}}}}$) and ethanol synthesis (${J}_{{{{{{\rm{epg}}}}}}}$ and ${J}_{{{{{{\rm{epx}}}}}}}$). Additionally, regulation exists during cellular substrate utilization, which includes the suppression of ethanol reassimilation by the sugars (glucose and xylose), inhibition of xylose consumption by ethanol^38^, and participation of the cofactor NAD+ in xylose utilization^42^. A detailed description of the model equations is provided in the Methods section. Upon parameter fitting, the model was shown to successfully produce the fermentation patterns of the consortium, including those of cell densities (OD_600_), glucose, xylose and ethanol in the presence of the compositional DOL (Supplementary Fig. 9) and temporal DOL (Supplementary Fig. 10). These results captured the characteristic behaviors of the corresponding experimental results (Figs. 3 and 4 and Supplementary Fig. 8). Detailed information of model parameter fitting is provided in Supplementary Method 1. The specific values of the model parameters are given in Supplementary Data 1. The sensitivity of the model parameters is evaluated in Supplementary Fig. 11.

### Model predictions and experimental validations of substrate co-fermentation

**Fig. 6:**
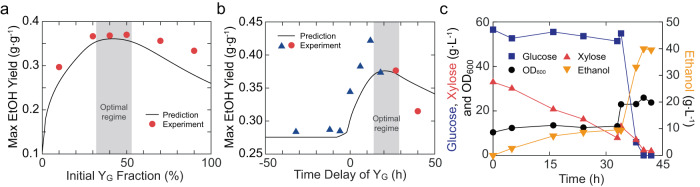
Model predictions and experimental validations for co-fermentation modulation. **a** Modeling-experiment comparison of the maximum ethanol yield by the consortium with compositional DOL. Line: model predictions; Circles: experimental data. **b** Modeling-experiment comparison of the maximal ethanol yield by the consortium under temporal DOL. Line: model predictions; Triangles: experimental data from the earlier fermentations in Supplementary Fig. 8; Circles: new experimental data from additional fermentations in Supplementary Fig. 13. **c** Temporal profiles of a plant hydrolysate fermentation by the engineered consortium that fulfills temporal DOL. The results of **c** are the mean of replicated experiments (*n* = 2). Source data are provided as a Source Data file.

With the demonstration of our model in capturing ecosystem behaviors, we set out to test if the model provides a predictive understanding for the fermentation optimization of the engineered consortium. We first used the model to simulate the co-fermentation of glucose and xylose, with their concentrations being around 70 g L^−1^ and 40 g L^−1^ respectively, under varied initial strain compositions (i.e., compositional DOL). Notably, the 7:4 substrate ratio was chosen to represent the concentrations of glucose and xylose in plant biomass hydrolysates but the specific values of the initial substrate concentrations in simulations were specified with the actual concentrations from experimental measurements. The model predicted that the maximal ethanol yield (i.e., maximal ethanol production normalized by substrate weight) of the co-fermentation follows a non-monotonic fashion (Fig. 6a, line). Specifically, the yield first increases monotonically, then reaches a plateau, and later declines slowly as the percentage of the glucose specialist varies from 0% to 100%. Notably, while there is a single maximum for the ethanol yield (0.361 g per g substrate) at 42% of the initial glucose specialist abundance, the ecosystem was predicted to reach at least 98% of the maximum (i.e., 0.354 g per g substrate) as long as the glucose specialist fraction falls within 32% to 53%. The predictions suggested that the plateau is flat and the ecosystem can reach a nearly optimal performance over a wide range of conditions. Encouragingly, the results from the corresponding fermentation experiments with Y_G2∆*ALD6*_ and Y_X3∆*ALD6*_ (Fig. 6a, circles, Supplementary Fig. 12) confirmed that different compositional DOL of the initial conditions indeed resulted in the alteration of glucose and xylose consumption as well as the maximal ethanol yield. Additionally, the results confirmed the existence of a plateau, including the initial Y_G_:Y_X_ ratios of 3:7, 4:6, and 5:5, for maximal ethanol yield and also confirmed the reduction of production with significantly unbalanced initial composition (e.g., 1:9 and 9:1) as predicted by the model. These results demonstrated once again that initial compositional DOL is a viable parameter for modulating community performance, and also confirmed the predictive capacity of the model.

Next, we used the model to reveal ethanol production under varied time delay between the inoculation of the two specialists (i.e., temporal DOL), which generated a non-monotonic profile of ethanol yield (Fig. 6b, line). This result captured the experimental characteristics of our previous fermentations for maximal ethanol yield (Fig. 6b, triangles). It also indicated that the ethanol yield has a plateau ($\ge$98% of the maximum (0.368 g per g substrate)) when the delay is between 14 to 29 hours (Fig. 6b, shaded region) and declines when the delay exceeds 29 h. To further confirm the modeling results, we conducted two additional fermentations. Consistent with the simulation, the ethanol yield remained high in the presence of 27 h of delay but was significantly decreased when the delay was increased to 40 hours (Fig. 6b, circles, Supplementary Fig. 13).

To further demonstrate the effectiveness of temporal DOL, we used the consortium to ferment a cellulosic hydrolysate containing 56.5 g L^−1^ of glucose and 35.2 g L^−1^ of xylose, instead of direct sugar mixtures. The xylose specialist Y_X3∆*ALD6*_ was inoculated ahead whereas the glucose specialist Y_G0∆*ALD6*_ was inoculated when the 10 g L^−1^ of xylose remained (Fig. 6c). As a result, both glucose and xylose were successfully depleted in 42 h, with a final 39.5 g L^−1^ of ethanol production. The result showed that temporal DOL provides the synthetic microbial consortium with a powerful capacity for the degradation of mixed sugars from the cellulosic hydrolysate.

### Glucose production

Aside from the test of DOL for enhancing microbial fermentation, we observed glucose accumulation during xylose consumption by Y_X2_, the xylose specialist strain with the deletion of *HXT1*, *HXT2*, and *GLK1*. Substantial amounts (1-2 g L^−1^) of glucose were accumulated from xylose fermentation by Y_X2_ and Y_X3_ (Fig. 1g and Supplementary Fig. 14d). To examine whether glucose can be also produced from glycerol by Y_X2_, Y_X2_ was cultured under glycerol conditions. Glucose production from glycerol was very low (~0.7 g L^−1^) with low cell densities (OD_600_ = 1) (Supplementary Fig. 14a), but substantial amounts (~1.5 g L^−1^) of glucose were produced with higher cell densities (OD_600_ = 10) (Supplementary Fig. 14b). These results suggested that, if glucose phosphorylation is blocked, yeast can produce glucose when other carbon sources such as xylose and glycerol are consumed. When xylose and glycerol were provided together as carbon sources, Y_X2_ produced 1.5 g L^−1^ of glucose, consuming both xylose and glycerol (Supplementary Fig. 14c). We hypothesized that glucose might be produced by dephosphorylation of glucose-6-phosphate (G6P) or glucose−1-phosphate (G1P) by endogenous phosphatases exhibiting promiscuous activities toward G6P or G1P^43^. As 2-deoxyglucose-6-phosphate has a similar structure to glucose-6-phosphate, we speculated that 2-deoxyglucose-6-phosphate phosphatase encoded by *DOG1* and *DOG2* might function as an endogenous phosphatase capable of producing glucose from either G6P or G1P^44^. Therefore, we overexpressed *DOG1* or *DOG2* under the control of a strong promoter in Y_X3_. The resulting strains (Y_X3_*DOG1* and Y_X3_*DOG2*) produced 30% more glucose (1.26 ± 0.01 g L^−1^ and 1.28 ± 0.02 g L^−1^ vs. 1.0 ± 0.09 g L^−1^) from xylose than Y_X3_. While Y_X3_*DOG1* and Y_X3_*DOG2* accumulated increased glucose titers, their xylose consumption and cell growth were reduced by 50% and 58%, respectively, as compared to Y_X3_ (Supplementary Fig. 14d–f). These results suggest that while promiscuous activity of DOG toward G6P or G1P might contribute to production of glucose from xylose, overexpression of DOG might be detrimental to xylose consumption and cell growth. Additional investigation will be necessary to elucidate the specific metabolic reactions and involved enzymes responsible for glucose production from xylose by *HXK1*, *HXK2*, and *GLK1* deleted strains^45–47^.

## Discussion

In this study, we combined experiments alongside mathematical modeling to elucidate the quantitative parameters and functional role of DOL in bioprocessing by microbial communities, using ethanol production from cellulosic hydrolysate containing glucose and xylose as an example. We constructed both glucose specialist and xylose specialist yeast strains that only ferment their associated sugars. By controlling initial cell density (i.e., compositional DOL) and inoculation timing (i.e., temporal DOL) of the glucose and xylose specialists, we demonstrated efficient and rapid fermentation of both mixed sugars at various concentrations and of cellulosic hydrolysate. In parallel to fermentation experiments, we constructed mathematical models describing fermentation behaviors of the synthetic microbial consortia composed of these two specialists. The models successfully captured quantitative characteristics and dynamic patterns of substrate utilization, predicting population growth as well as ethanol production. Experimental validation of predictions for maximal product generation further confirmed the accuracy of our models.

Energy crop variety, pretreatment method, and usage of enzymatic hydrolysis leads to wide variation in glucose and xylose concentration of cellulosic hydrolysate. For example, Cheng et al. reported production of cellulosic hydrolysate containing 76.31–123.47 g L^−1^ of glucose and 34.30–67.84 g L^−1^ of xylose from a bioenergy sorghum plant using chemical-free pretreatment and enzymatic hydrolysis^48^, and Lau et al. reported production of cellulosic hydrolysate containing 57.5–68.0 g L^−1^ of glucose and 28.1–39.8 g L^−1^ of xylose from ammonia fiber expansion (AFEX)-treated corn stover^49^. Because of this sugar concentration variation, single strain fermentation of cellulosic hydrolysates often requires strain optimization for sugars in the specific hydrolysate used to maximize efficiency. As a solution, we hypothesized that synthetic microbial consortia might be better suited to cellulosic hydrolysate fermentation, as wild microbial communities still thrive in environments with varied nutrients^50^. In addition, rather than attempt additional strain manipulation to optimize for sugar concentrations, the temporal and compositional DOL unique to this synthetic consortium method might more easily maximize hydrolysate fermentation efficiency. Indeed, the experiments conducted with our synthetic consortia bore out these hypotheses with rapid and efficient fermentation of mixed sugars at various concentrations in both media and cellulosic hydrolysate. Furthermore, fermentation tunability through temporal and compositional DOL was demonstrated through the combination of these experiments with mathematical modeling, showing that fermentation behavior can be predicted and maximized using said models.

Synthetic microbial consortia of engineered *E. coli* strains for biofuel and chemical production from mixtures of glucose and xylose have been reported. Eiteman et al. demonstrated co-consumption of glucose and xylose by co-culturing of glucose and xylose specialist *E. coli* strains^15^. Flores et al. also used *E. coli* specialist strain co-culturing of glucose and xylose mixtures, producing D-lactate and succinate. Nonetheless, *E. coli*-based synthetic microbial consortia have not been used to ferment lignocellulosic hydrolysates, which contain varying levels of glucose, xylose, and acetate as well as various fermentation inhibitors. Although E. coli-based synthetic microbial consortia have been used to fermentation lignocellulosic hydrolysates, due to the toxicity of hydrolysate to *E. coli*-based microbial consortia pH was maintained higher (pH 7.0) and fermented xylose concentration was lower (15 g L^−1^) than in this study^51^. Our synthetic microbial consortium was able to successfully ferment cellulosic hydrolysate, as the yeast strains used exhibit much higher tolerance of fermentation inhibitors than engineered *E. coli* strains typically can.

Simultaneous consumption of mixed sugars by a single microorganism has been proposed as a strategy for efficient production of biofuels and chemicals from cellulosic hydrolysate. To this end, adaptive laboratory evolution^52^, identification and engineering of xylose transporters lacking glucose inhibition^32,35^, and co-consumption of cellobiose and xylose has been reported^53^. While simultaneous co-consumption of glucose and xylose can be achieved through this path, it has only been achieved by reducing glucose consumption rates below xylose consumption rates. As a result, overall fermentation rates are not drastically improved even though glucose and xylose are simultaneously consumed. In this study, we proposed and validated a practical alternative strategy where consumption order of glucose and xylose is altered to enhance overall fermentation rates of mixed sugars and cellulosic hydrolysates. We showed the highest ethanol productivity and yield among the studies that attempted mixed sugar fermentation using microbial consortium (Supplementary Table 1). Recent efforts have showed that altering the initial community ratio or the timing of inoculation modulates community dynamics and composition;^54,55^ here, this study demonstrates that a combination of these experimental strategies with mathematical modeling serves as an effective strategy for the optimization of fermentation characteristics and product synthesis. By enabling consumption of xylose ahead of glucose by temporal DOL of synthetic consortia, we can expect multiple benefits towards producing cellulosic ethanol: (i) minimization of ethanol inhibition on xylose consumption, (ii) operation of the less efficient xylose fermentation first, (iii) elimination of metabolic burdens from engineering of mixed sugar consumption pathways in a single strain, and (iv) the conversion rates of each sugar can be individually modulated to optimize overall process in producing non-growth product^15^. In this study, this “alignment” of consumption rates was exacted by inoculating the culture at different times, thereby allowing each strain to reach a desired cell density prior to switching to a non-growth production phase. However, other means might be available to align consumption rates in other circumstances, for example, using differential inoculation densities or introducing genetic modifications which affect growth rates.

Further investigations might be necessary to apply our synthetic microbial consortia for industrial fermentation especially under acidic conditions with fermentation inhibitors. Our study clearly demonstrated the advantages of DOL for agile control and optimization of microbial fermentation. We envision that similar strategies can be undertaken to produce biofuels and chemicals from cellulosic hydrolysates. We presented a set of highly engineered yeast strains with marvelous fermentation phenotypes in terms of sugar utilization and corresponding mathematical models that can be utilized for the efficient and rapid conversion of cellulosic hydrolysates to value-added products.

## Methods

### Construction of strains and plasmids

All strains used in this paper are listed in Supplementary Table 2. The plasmids, primers, and guide RNA (gRNA) target sequences used in this paper are listed in Supplementary Tables 3, 4 and 5 respectively. *E. coli* Top10 strain was used for amplification and manipulation of all plasmids. If the plasmid was present, *E. coli* strains were cultured with ampicillin (100 µg mL^−1^) in Luria Bertani (LB) media at 37 °C, 250 rpm. To make the expression vector, the coding sequences (CDS) were synthesized at IDT (Integrated DNA Technology, IA, USA). Empty expression vector and CDS were opened and amplified using Phusion polymerase (New England Biolabs, MA, USA), respectively. Ligation independent cloning method was used to combine two fragments^56^.

To integrate expression cassettes into yeast chromosomes or to delete genes, the CRISPR/Cas9 system was used. Cas9-NAT plasmid (Addgene #64329) was transformed before Cas9-Based genetic manipulation. Guide RNA targeting a specific gene was designed using the gRNA design tool (www.atum.bio/eCommerce/cas9/input) and manufactured using the standard molecular biology method. Intergenic sites of CS5 (on Chr XV) CS6 (on Chr VII), CS8 (on Chr XVI) were used for expression cassette integration (Supplementary Table 5)^57,58^. Donor DNA fragments were co-transformed with a guide RNA targeting a specific gene or intergenic site in *S. cerevisiae* expressing Cas9. Cells genetically engineered by Cas9 were selected with 120 µg mL^−1^ nourseothricin and 300 µg mL^−1^ hygromycin B (Merck, Germany) or G418 (Genview, FL, USA) depending on the gRNA used. Genetically engineered positive colonies were confirmed by diagnostic PCR, which amplifies around the target sequence.

### Directed evolution of a sugar transporter

Random mutagenesis of *At*Sweet1 was performed using mutagenesis primers (Supplementary Table 4) using a Genemorph Mutazyme II kit (Agilent Technologies, Santa Cruz, CA, USA). Following supplier directions, libraries using low (0–4.5 mutations per kb), medium (4.5–9 mutations per kb), and high (9–16 mutations per kb) mutagenesis rates were cloned into p42K-GPD1p-CYC1t at a library size of 10^−5^. These libraries were then transformed into *S. cerevisiae* SR8D8 and plated on a YP medium with 20 g L^−1^ xylose and 200 µg mL^−1^ geneticin. Using a wild-type transformation for comparison, 6–15 large-size colonies were selected from each library transformation plate for a total of approximately 50 mutants per transporter per round. A growth rate measurement against control on a Bioscreen C (Growth Curves USA, Piscataway, NJ) served as a quantitative screen. The vectors from those promising mutants were isolated, sequenced, and retransformed into *S. cerevisiae* SR8D8. A second growth rate measurement against control confirmed that the growth rate increase was due to the mutant transporter and not the background adaptation of the host strain.

### Yeast cell cultures

For comparison of sugar consumption rate between specialist strains, engineered strains inoculated from glycerol stock were cultured in 5 mL YP media containing 20 g L^−1^ glucose or xylose at 1% (Vol Vol^−1^) final inoculation concentration at 30  ^°^C, 250 rpm for 48 h. In the main culture, each strain was cultured in YP media containing glucose or xylose or a mixture of glucose and xylose at a final concentration of OD_600_ 1 at 30 °C, 250 rpm. For yeast cell culture for ethanol fermentation, each strain was precultured as described above. The precultured strains were inoculated in 25 mL of YP media having various glucose and xylose concentrations at a final concentration of OD_600_ 1 and incubated at 100 rpm and 30 °C. A bioenergy sorghum hydrolysate produced by hydrothermal pretreatment and hydrolysis by cellulases was used for fermentation experiments. The initial pH of the hydrolysate was pH 4.8 and sodium hydroxide (Sigma-Aldrich, St. Louis, MO, USA) was added to increase the pH to 5.9. Solid residues in the hydrolysates were removed by centrifugation at 12298 g for 5 min and the supernatant was sterilized using 0.22 µm PES membrane filter (Merck Millipore, Darmstadt, Germany) before fermentation.

### Analytical methods

To determine the cell density, the absorbance at 600 nm was measured using a spectrophotometer. Dried cell weight (DCW) was calculated using the equation of OD_600_ and DCW obtained in advance. Glucose, xylose, and ethanol concentrations inside the media were analyzed using an Agilent 1200 HPLC equipped with a refractive index detector (Agilent, CA, USA) and Rezex ROA-Organic Acid H^+^ 8% column (Phenomenex, CA, USA). The mobile phase of HPLC is 0.005 N H_2_SO_4_, and the flow rate and column temperature were maintained at 0.6 mL min^−1^ and 50  ^°^C, respectively.

### Mathematical model

Under the assumption that the specialists are well-mixed and identical among their individual populations, the fermentation kinetics of the consortium can be mathematically described with the consideration of the extracellular concentrations of glucose ($G$), xylose ($X$) and ethanol ($E$), the single-cell precursor pools (${R}_{{{{{{\rm{g}}}}}}}$ and ${R}_{{{{{{\rm{x}}}}}}}$) and the populations of the glucose and xylose specialists (${N}_{{{{{{\rm{g}}}}}}}$ and ${N}_{{{{{{\rm{x}}}}}}}$) as follows:

1 $$\frac{{dG}}{{dt}}=-{J}_{{{{{{\rm{g}}}}}}}\cdot {N}_{{{{{{\rm{g}}}}}}}$$

2 $$\frac{{dX}}{{dt}}=-{J}_{{{{{{\rm{x}}}}}}}\cdot {N}_{{{{{{\rm{x}}}}}}}$$

3 $$\,\frac{{dE}}{{dt}}={\beta }_{{{{{{\rm{epg}}}}}}}{\cdot J}_{{{{{{\rm{epg}}}}}}}\cdot {N}_{{{{{{\rm{g}}}}}}}+{\beta }_{{{{{{\rm{epx}}}}}}}\cdot {J}_{{{{{{\rm{epx}}}}}}}\cdot {N}_{{{{{{\rm{x}}}}}}}-{J}_{{{{{{\rm{ecg}}}}}}}\cdot {N}_{{{{{{\rm{g}}}}}}}-{J}_{{{{{{\rm{ecx}}}}}}}\cdot {N}_{{{{{{\rm{x}}}}}}}$$

4 $$\frac{d{R}_{{{{{{\rm{g}}}}}}}}{{dt}}={\beta }_{{{{{{\rm{g}}}}}}}{\cdot J}_{{{{{{\rm{g}}}}}}}+{\beta }_{{{{{{\rm{ecg}}}}}}}\cdot {J}_{{{{{{\rm{ecg}}}}}}}-{J}_{{{{{{\rm{ng}}}}}}}-{J}_{{{{{{\rm{epg}}}}}}}-{J}_{{{{{{\rm{mg}}}}}}}-{\gamma }_{{{{{{\rm{g}}}}}}}\cdot {J}_{{{{{{\rm{ng}}}}}}}\cdot {R}_{{{{{{\rm{g}}}}}}}$$

5 $$\frac{d{R}_{{{{{{\rm{x}}}}}}}}{{dt}}={\beta }_{{{{{{\rm{x}}}}}}}\cdot {J}_{{{{{{\rm{x}}}}}}}+{\beta }_{{{{{{\rm{ecx}}}}}}}\cdot {J}_{{{{{{\rm{ecx}}}}}}}-{J}_{{{{{{\rm{nx}}}}}}}-{J}_{{{{{{\rm{epx}}}}}}}-{J}_{{{{{{\rm{mx}}}}}}}-{{\gamma }_{{{{{{\rm{x}}}}}}}\cdot J}_{{{{{{\rm{nx}}}}}}}{\cdot R}_{{{{{{\rm{x}}}}}}}$$

6 $$\frac{d{N}_{{{{{{\rm{g}}}}}}}}{{dt}}={{\gamma }_{{{{{{\rm{g}}}}}}}\cdot J}_{{{{{{\rm{ng}}}}}}}\cdot {N}_{{{{{{\rm{g}}}}}}}$$

7 $$\frac{d{N}_{{{{{{\rm{x}}}}}}}}{{dt}}={{\gamma }_{{{{{{\rm{x}}}}}}}\cdot J}_{{{{{{\rm{nx}}}}}}}\cdot {N}_{{{{{{\rm{x}}}}}}}$$

where the terms ${J}_{[\ldots ]}$ refer to single-cell carbon fluxes, the factors ${\beta }_{\left[\ldots \right]}$ are the conversion coefficients between different molecules, and ${\gamma }_{{{{{{\rm{g}}}}}}}$ and ${\gamma }_{{{{{{\rm{x}}}}}}}$ are the conversion coefficients between the fluxes toward biomass and actual growth rates.

Specifically, the glucose utilization follows a Monod equation form as

8 $${J}_{{{{{{\rm{g}}}}}}}=\frac{{\alpha }_{{{{{{\rm{g}}}}}}}G}{{K}_{{{{{{\rm{g}}}}}}}+G}$$

where ${\alpha }_{{{{{{\rm{g}}}}}}}$ and ${K}_{{{{{{\rm{g}}}}}}}$ are the maximal rate and half-velocity constant of glucose consumption.

Different from glucose utilization, the utilization of xylose was modeled as

9 $${J}_{{{{{{\rm{x}}}}}}}=\frac{1}{1+{k}_{{{{{{\rm{x}}}}}}}E}\cdot \frac{{C}_{{{{{{\rm{x}}}}}}}}{{K}_{{{{{{\rm{C}}}}}}}+{C}_{{{{{{\rm{x}}}}}}}}\cdot \frac{{\alpha }_{{{{{{\rm{x}}}}}}}X}{{K}_{{{{{{\rm{x}}}}}}}+X}$$

where ${\alpha }_{{{{{{\rm{x}}}}}}}$ and ${K}_{{{{{{\rm{x}}}}}}}$ are the maximal rate and half-velocity constant separately. Here, the first term describes the inhibition of xylose consumption by ethanol and the second term describe the dependence of the cofactor NAD^+^ (${C}_{{{{{{\rm{x}}}}}}}$)^42^, with the coefficients ${k}_{{{{{{\rm{x}}}}}}}$ and ${K}_{{{{{{\rm{C}}}}}}}$ characterizing the inhibitory effect and the half-velocity constant of NAD^+^ utilization.

The precursor-to-ethanol fluxes were modeled as

10 $${J}_{{{{{{\rm{epg}}}}}}}=\frac{{\alpha }_{{{{{{\rm{epg}}}}}}}{R}_{{{{{{\rm{g}}}}}}}}{{K}_{{{{{{\rm{epg}}}}}}}+{R}_{{{{{{\rm{g}}}}}}}}$$

and

11 $${J}_{{{{{{\rm{epx}}}}}}}=\frac{{\alpha }_{{{{{{\rm{epx}}}}}}}{R}_{{{{{{\rm{x}}}}}}}}{{K}_{{{{{{\rm{epx}}}}}}}+{R}_{{{{{{\rm{x}}}}}}}}$$

for glucose and xylose, respectively,where ${\alpha }_{{{{{{\rm{epg}}}}}}}$, ${K}_{{{{{{\rm{epg}}}}}}}$, ${\alpha }_{{{{{{\rm{epx}}}}}}}$ and ${K}_{{{{{{\rm{epx}}}}}}}$ are the corresponding maximal rates and half-velocity constants.

The reassimilation of ethanol by the glucose and xylose specialists takes the form of

12 $${J}_{{{{{{\rm{ecg}}}}}}}=\frac{1}{1+{k}_{{{{{{\rm{ecg}}}}}}}G}\cdot \frac{{\alpha }_{{{{{{\rm{ecg}}}}}}}E}{{K}_{{{{{{\rm{ecg}}}}}}}+E}$$

and

13 $${J}_{{{{{{\rm{ecx}}}}}}}=\frac{1}{1+{k}_{{{{{{\rm{ecx}}}}}}}X}\cdot \frac{{\alpha }_{{{{{{\rm{ecx}}}}}}}E}{{K}_{{{{{{\rm{ecx}}}}}}}+E}$$

respectively,whereby the first term in each expressions describes the catabolic inhibition by the corresponding sugar and the second term refers to the specific characteristics of ethanol consumption. Here, ${k}_{{{{{{\rm{ecx}}}}}}}$ and ${k}_{{{{{{\rm{ecg}}}}}}}$ are the coefficients defining the inhibition of the sugars on ethanol reutilization whereas ${\alpha }_{{{{{{\rm{ecg}}}}}}}$, ${K}_{{{{{{\rm{ecg}}}}}}}$, ${\alpha }_{{{{{{\rm{ecx}}}}}}}$ and ${K}_{{{{{{\rm{ecx}}}}}}}$ are the corresponding maximal rates and half-velocity constants. The fluxes to biomass growth and maintenance were modeled as

14 $${J}_{{{{{{\rm{ng}}}}}}}=\frac{{\alpha }_{{{{{{\rm{ng}}}}}}}{R}_{{{{{{\rm{g}}}}}}}}{{K}_{{{{{{\rm{ng}}}}}}}+{R}_{{{{{{\rm{g}}}}}}}}$$

and

15 $${J}_{{{{{{\rm{mg}}}}}}}=\frac{{\alpha }_{{{{{{\rm{mg}}}}}}}{R}_{{{{{{\rm{g}}}}}}}}{{K}_{{{{{{\rm{mg}}}}}}}+{R}_{{{{{{\rm{g}}}}}}}}$$

for the glucose specialist and

16 $${J}_{{{{{{\bf{nx}}}}}}}=\frac{{\alpha }_{{{{{{\rm{nx}}}}}}}{R}_{{{{{{\rm{x}}}}}}}}{{K}_{{{{{{\rm{nx}}}}}}}+{R}_{{{{{{\rm{x}}}}}}}}$$

and

17 $${J}_{{{{{{\rm{mx}}}}}}}=\frac{{\alpha }_{{{{{{\rm{mx}}}}}}}{R}_{{{{{{\rm{x}}}}}}}}{{K}_{{{{{{\rm{mx}}}}}}}+{R}_{{{{{{\rm{x}}}}}}}}$$

for the xylose specialist where ${\alpha }_{{{{{{\rm{ng}}}}}}}$, ${K}_{{{{{{\rm{ng}}}}}}}$, ${\alpha }_{{{{{{\rm{mg}}}}}}}$, ${K}_{{{{{{\rm{mg}}}}}}}$, ${\alpha }_{{{{{{\rm{nx}}}}}}}$, ${K}_{{{{{{\rm{nx}}}}}}}$, ${\alpha }_{{{{{{\rm{mx}}}}}}}$ and ${K}_{{{{{{\rm{mx}}}}}}}$ are the corresponding maximal rates and half-velocity constants.

For the consortium, dissolved oxygen participates in cellular metabolism, contributing to cellular cofactor balance^59^. Thus, the model also included the kinetics of extracellular oxygen availability ($O$) as

18 $$\frac{{dO}}{{dt}}={v}_{{{{{{\rm{b}}}}}}}-{J}_{{{{{{\rm{og}}}}}}}{N}_{{{{{{\rm{g}}}}}}}-{{J}_{{{{{{\rm{ox}}}}}}}N}_{{{{{{\rm{x}}}}}}}$$

which includes its external supplementation in the microaerobic condition with a constant rate ${v}_{{{{{{\rm{b}}}}}}}$ and consumption by the glucose and xylose specialists with a rate of

19 $${J}_{{{{{{\rm{og}}}}}}}={J}_{{{{{{\rm{ox}}}}}}}=\frac{{\alpha }_{{{{{{\rm{o}}}}}}}O}{{K}_{{{{{{\rm{o}}}}}}}+O}$$

where ${\alpha }_{{{{{{\rm{o}}}}}}}$ is the maximal rate and ${K}_{{{{{{\rm{o}}}}}}}$ is the half-velocity constant. For the glucose specialist, its cofactor balance was assumed to be maintained throughout the fermentation via cellular innate homeostasis mechanism. For the xylose specialist, due to the imbalanced NAD^+^ demand for xylose consumption, its NAD^+^ concentration (${C}_{{{{{{\rm{x}}}}}}}$) was explicitly modeled as

20 $$\frac{d{C}_{{{{{{\rm{x}}}}}}}}{{dt}}={\beta }_{{{{{{\rm{o}}}}}}}\cdot {J}_{{{{{{\rm{ox}}}}}}}-{J}_{{{{{{\rm{bc}}}}}}}-{k}_{{{{{{\rm{xc}}}}}}}\cdot {J}_{{{{{{\rm{x}}}}}}}-{{\gamma }_{{{{{{\rm{x}}}}}}}\cdot J}_{{{{{{\rm{nx}}}}}}}\cdot {C}_{{{{{{\rm{x}}}}}}}$$

which involves regeneration mediated by oxygen^59^, basic consumption for cellular activity, consumption during xylose utilization, and growth-associated decay respectively. Here, ${\beta }_{{{{{{\rm{o}}}}}}}$ is the coefficient of NAD^+^ regeneration, ${k}_{{{{{{\rm{xc}}}}}}}$ measures the amount of NAD^+^ used in xylose utilization, and the basic consumption ${J}_{{{{{{\rm{bc}}}}}}}$ takes the form of

21 $${J}_{{{{{{\rm{bc}}}}}}}=\frac{{\alpha }_{{{{{{\rm{bc}}}}}}}{C}_{{{{{{\rm{x}}}}}}}}{{K}_{{{{{{\rm{bc}}}}}}}+{C}_{{{{{{\rm{x}}}}}}}}$$

where ${\alpha }_{{{{{{\rm{bc}}}}}}}$ and ${K}_{{{{{{\rm{bc}}}}}}}$ are the maximal rate and half-velocity constant.

### Reporting summary

Further information on research design is available in the Nature Portfolio Reporting Summary linked to this article.

### Supplementary information

Supplementary Information

Description of Additional Supplementary Files

Supplementary Data 1

Reporting Summary

### Source data

Source Data

## Data Availability

Data supporting the findings of this work are available within the paper and its Supplementary Information files. A reporting summary for this article is available as a Supplementary Information file. Variables and parameters of the mathematical model were provided in Supplementary Data 1. All other data are available from the corresponding author upon request. Source data are provided in this paper.

## Code availability

Custom-built MATLAB code is available at Github https://github.com/Lulabcodes/YGXCoC.
