# Supplementary material for: Compositional and temporal division of labor modulates mixed sugar fermentation by an engineered yeast consortium
Source: Nat Commun. 2024 Jan 26;15:781. doi: 10.1038/s41467-024-45011-w (PMC10817915; doi:10.1038/s41467-024-45011-w)
Supplement: Supplementary file 1 — Supplementary Information [file 41467_2024_45011_MOESM1_ESM.pdf]

Compositional and temporal division of labor modulates mixed sugar fermentation by an engineered yeast consortium

Shin *et al.*

Supplementary Method 1. Computational methods

The code for computational modeling was written in MATLAB 2021b. The model equations were solved using *ode15s* with the non-negative setting. The absolute error tolerance was set as 1e-7 and the relative error tolerance was set as 1e-5.

The initial conditions were selected based on the following guidelines: (1) For model fitting, cellular optical densities (ODs) were set based on the experimental measurements of total ODs and the designed strain composition ratios whereas the sugar concentrations were set as the experimentally measured initial values. Notably, for a substrate that was not consumed until a corresponding strain was added at a specific time point, its concentration was set as the average of measurements taking from the beginning of the experiment to that specific time point. (2) For model prediction, the initial conditions were set based on the averaged initial conditions of the relevant experimental systems. Specifically, 78 g L^{-1} glucose, 47 g L^{-1} xylose and total OD of 10 were used in Fig. 6a; 71 g L^{-1} glucose, 41 g L^{-1} xylose and OD of 9 for each strain were used in Fig. 6b. (3) Precursor concentrations were initially set as 0. For the NAD^+ in xylose specialist and oxygen in environment, their initial concentrations were normalized as 1.

For each specialist, its precursor outfluxes to maintenance, cell growth and ethanol production were all described in a Hill function form. To reflect the fact that cells may have different priorities for these fluxes with the one for maintenance being superior to the others, the half-velocity constants of the maintenance fluxes (K_{mg} and K_{mx}) were set to be 1e-6 to ensure that they are much smaller than those for growth and ethanol production (K_{ng}, K_{epg}, K_{nx} and K_{epx}). As the three strains (two glucose specialists and one xylose specialist) possess the same modules for the utilization of precursor for cell growth, maintenance and ethanol production, we assumed the parameters of these shared modules were identical among the strains.

In our study, we used a least-square method to estimate the model parameters. The model parameters $\boldsymbol{\theta}$ were estimated with a training dataset, that includes both the compositional (Fig. 3) and temporal DOL (Fig. 4d-f and Supplementary Fig. 8) data, by minimizing the objective function

$$f(\boldsymbol{\theta}) = \sum_{j}^{m}\sum_{i}^{n}\left(\frac{y_{ij} - \tilde{y}_{ij}(\boldsymbol{\theta})}{h_j}\right)^2 \quad \textbf{(1)}$$

where y_{ij} is the *i*-th time-course data of the *j*-th variable, with *j*=1,2,3,4 corresponds to the concentration of glucose, xylose and ethanol and OD of the culture in experiments respectively, and $\tilde{y}_{ij}(\boldsymbol{\theta})$ is the corresponding model simulation. For each variable, the errors are normalized by h_j, the mid-range value of the experimental data (39 g L^{-1}, 28 g L^{-1}, 24 g L^{-1} and 22 for glucose, xylose, ethanol and OD, respectively). The optimization problem was solved in MATLAB using *fmincon*. The detailed parameters are provided in the Supplementary Data File. The confidence intervals of the parameters were estimated by MATLAB function *nlparci* utilizing the information of residuals and the derivatives of the model output with respect to the parameters near the parameters we inferred.

To quantitatively illustrate the quality of the model parameters, we calculated the root-mean-square deviation (RMSD) between model fitting and experimental data based on the weighted errors. Mathematically, RMSD is defined as

$$\mathrm{RMSD} = \sqrt{\frac{\sum_j^m \sum_i^n \left(\frac{y_{ij} - \tilde{y}_{ij}}{h_j}\right)^2}{mn - p - q}} \tag{2}$$

where $m = 4$ is the number of variables, $n = 150$ is the total number of time points, $p = 29$ is the number of parameters, $q = 48$ is the number of model outputs at time $t = 0$ which are fixed during fitting. For our model, $\mathrm{RMSD} = 0.13$, suggesting that the average level of model error is around 13% of the mid-range value of experimental measurements.

To evaluate the sensitivity of the model parameters, we showed the change of RMSD upon the variation of each parameter, which is detailed in Supplementary Fig. 11.

Supplementary Table 1. Ethanol production reported by this and previous studies.

**Microorganism**	***E. coli***	***S. cerevisiae***	***S. cerevisiae***	***S. cerevisiae***	***S. cerevisiae***
Consumed sugars	Glucose (9 g L^{-1}), Xylose (5 g L^{-1})	Cellobiose (60 g L^{-1}), Xylose (20 g L^{-1})	Glucose (17.5 g L^{-1}), Xylose (10 g L^{-1}), Arabinose (5 g L^{-1}).	Glucose (80 g L^{-1}), Xylose (45 g L^{-1})	Glucose (70 g L^{-1}), Xylose (40 g L^{-1})
Produced ethanol	2 g L^{-1}	32 g L^{-1}	12.5 g L^{-1}	46.9 g L^{-1}	48.4 g L^{-1}
Ethanol productivity	1 g L^{-1} h^{-1}	1.33 g L^{-1} h^{-1}	0.35 g L^{-1} h^{-1}	0.49 g L^{-1} h^{-1}	1.79 g L^{-1} h^{-1}
Reference	[1]	[2]	[3]	In this study (Compositional DOL: Supplementary figure 12C)	In this study (Temporal DOL: Figure 4f)

Supplementary Table 2. The strains used in this study.

Strain	Description	Reference
SR8	Xylose-consuming engineered *S. cerevisiae*	[4]
$SR8_{\Delta ALD6}$	SR8 $_{\Delta ALD6}$	This study
SR8D8	SR8 Δ *HXT1-7* Δ *GAL2*	[5]
Y_{X1}	SR8D8 CS5::LSNF CS6::mRuby2	This study
Y_{X2}	SR8D8 Δ *HXK1* Δ *HXK2* Δ *GLK1* CS5::LSNF CS6::mRuby2	This study
Y_{X3}	SR8D8 Δ *HXK1* Δ *HXK2* Δ *GLK1* CS5::LSNF CS6::mRuby2 CS8::LSNF	This study
$Y_{X3\Delta ALD6}$	SR8D8 Δ *ALD6* Δ *HXK1* Δ *HXK2* Δ GLK1 CS5::LSNF CS6::mRuby2 CS8::LSNF	This study
Y_{G0}	D452-2 CS5::GFP	This study
$Y_{G0\ \Delta ALD6}$	D452-2 Δ ALD6 CS5::GFP	This study
Y_{G1}	SR8D8 CS5::*At*Sweet1* CS6::GFP	This study
Y_{G2}	SR8D8 CS5:: *At*Sweet1 CS6::GFP	This study
$Y_{G2\Delta ALD6}$	SR8D8 Δ *ALD6* CS5:: *At*Sweet1 CS6::GFP	This study

Supplementary Table 3. The plasmids used in this study.

Name	Description	Reference
pRS426	Empty expression cassette with *TDH3* promoter and *CYC1* terminator, 2 μ origin, and Amp^R	[6]
pRS42K	2 μ origin, *KanMX* for G418 resistance	EUROSCARF
pRS42H	2 μ origin, *hph* for hygromycin B resistance	EUROSCARF
pRS426-*At*Sweet1	Expression cassette of *At*Sweet1 (Sugar transporter originated from *Arabidopsis thaliana*) with *TDH3* promoter and *CYC1* terminator, 2 μ origin, and Amp^R	[7]
pRS426-*At*Sweet1*	The expression cassette of *At*Sweet1* (*At*Sweet1 mutant V69M, G75A obtained from evolutionary engineering) with *TDH3* promoter and *CYC1* terminator, 2 μ origin, and Amp^R	This study
pRS426-D2NF	The expression cassette of D2NF (N370F mutant sugar transporter originated from *Lypomyces starkeyi*) with *TDH3* promoter and *CYC1* terminator, 2 μ origin, and Amp^R	[7]
pRS426-mRuBy2	The expression cassette of mRuby2 with *TDH3* promoter, *CYC1* terminator, 2 μ origin, and Amp^R	This study
pRS426-GFP	The expression cassette of GFP with *TDH3* promoter, *CYC1* terminator, 2 μ origin, and Amp^R	This study
pRS42K-CS5	pRS42K, gRNA targeting for the intergenic site on chromosome XV	[8]
pRS42H-CS6	pRS42H, gRNA targeting for the intergenic site on chromosome VII	[9]
pRS42H-CS8	pRS42H, gRNA targeting for the intergenic site on chromosome XVI	[9]
pRS42K-*HXK1*	pRS42K, gRNA targeting for *HXK1*	This study
pRS42H-*HXK2*	pRS42H, gRNA targeting for *HXK2*	This study
pRS42K-*GLK1*	pRS42K, gRNA targeting for *GLK1*	This study
pRS42H-*ALD6*	pRS42H, gRNA targeting for *ALD6*	This study

Supplementary Table 4. The primers used in this study.

Name	Sequence (5′-3′)
pRS426-Vector FW	atcaagcttatcgataccgtcgacctcgag
pRS426-Vector BW	gatccactagttctagaatccgtcgaaactaagttctggt
mRuby2 FW	tagaactagtggatcatggtgtctaagggcgaagagctgatcaag
mRuby2 BW	atcgataagcttgatttacttgtacagctcgtccatcccaccac
GFP FW	tagaactagtggatcatgtctaaaggtgaagaattattcactggtgttgtcccaatt
GFP BW	atcgataagcttgatttatttgtacaattcatccataccatgggtaataccagcagca
*At*Sweet1 FW	attgcggatccaaaaaaatgaacatcgctcacactatcttcg
*At*Sweet1 BW	attgcctcgagttaaacttgaaggtcttgctttccatta
HXK1 gRNA FW	atgggtgtgattttcggtacgttttagagctagaaatagcaag
HXK1 gRNA BW	gtaccgaaaatcacacccatgatcatttatctttcactgcgga
HXK2 gRNA FW	gacttgttccaaaatgagttgttttagagctagaaatagcaag
HXK2 gRNA BW	aactcattttggaacaagtcgatcatttatctttcactgcgga
GLK1 gRNA FW	cgaggttacccccgagaagcgttttagagctagaaatagcaag
GLK1 gRNA BW	gcttctcgggggtaacctcggatcatttatctttcactgcgga
ALD6 gRNA FW	ttagagccaatcggtgtctggttttagagctagaaatagcaag
ALD6 gRNA BW	cagacaccgattggctctaagatcatttatctttcactgcgga

Supplementary Table 5. The gRNA target sequences used in this study.

**Name**	**Sequence (5′-3′)**
HXK1	ATGGGTGTGATTTTCGGTAC
HXK2	GACTTGTTCCAAAATGAGTT
GLK1	CGAGGTTACCCCCGAGAAGC
ALD6	TTAGAGCCAATCGGTGTCTG
CS5	TCTTTCTGTGCAACTACCAG
CS6	GATACTTATCATTAAGAAAA
CS8	TGATTCAATCATTCTTATTG

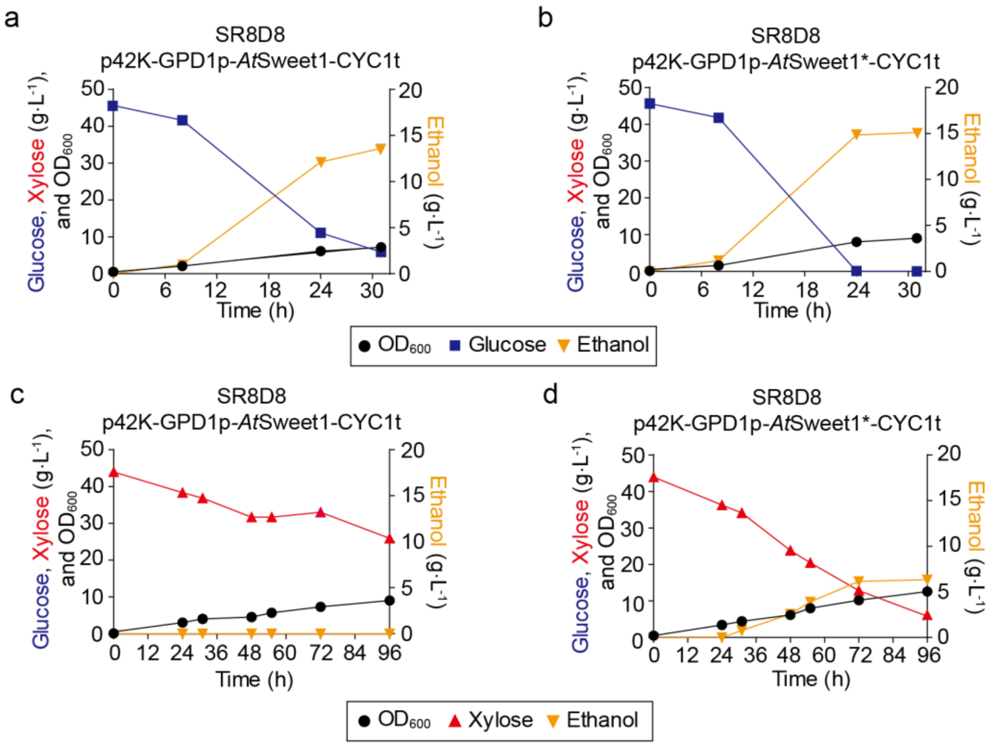

Supplementary Figure 1. The sugar consumption rate of *At*Sweet1 and *At*Sweet1*. Glucose consumption profile of (a) SR8D8 p42K-GPD1p-*At*Sweet1-CYC1t and (b) SR8D8 p42K-GPD1p-*At*Sweet1*-CYC1t. Xylose consumption profile of (c) SR8D8 p42K-GPD1p-*At*Sweet1-CYC1t and (d) SR8D8 p42K-GPD1p-*At*Sweet1*-CYC1t. Results are the mean of replicated experiments (n=2). Source data are provided as a Source Data file.

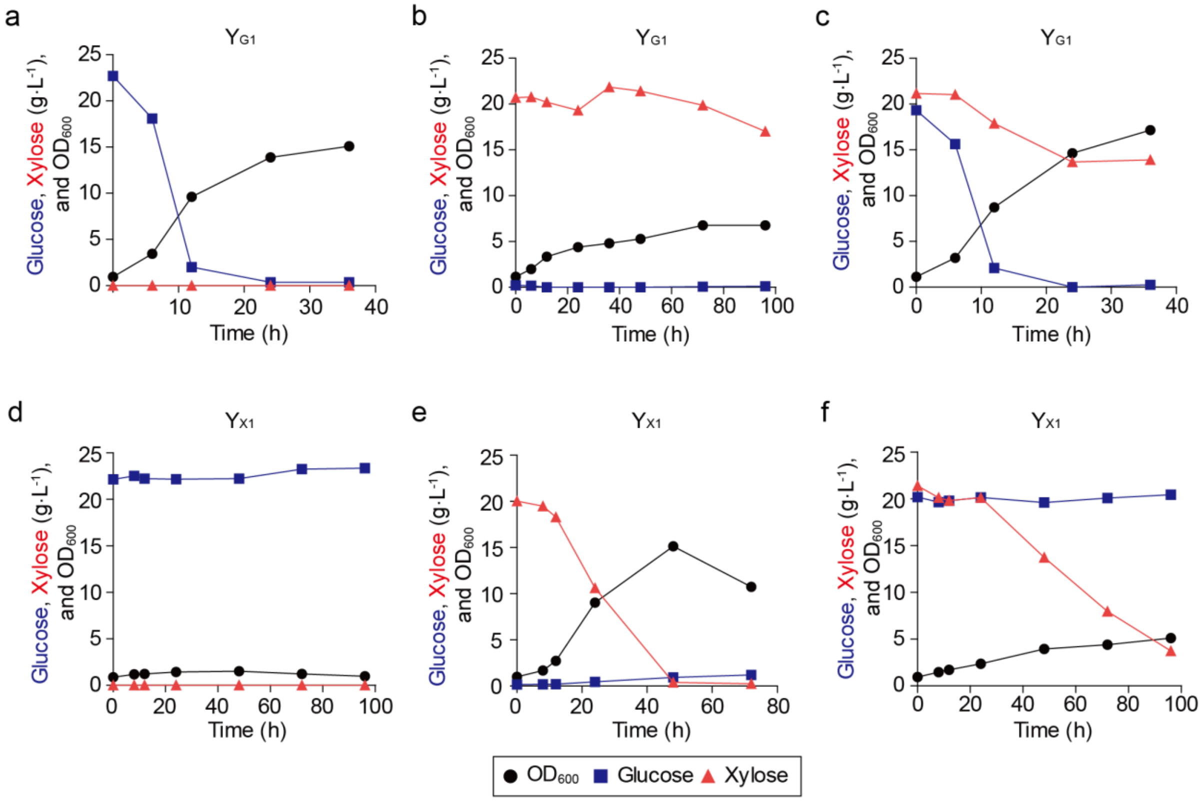

Supplementary Figure 2. Consumption of glucose and xylose by Y_{G1} and Y_{X1}. Sugar consumption profile of Y_{G1} in (a) YPglucose, (b) YPxylose, and (c) YPglucose+xylose. Sugar consumption profile of Y_{X1} in (d) YPglucose, (e) YPxylose, and (f) YPglucose+xylose. Results are the mean of replicated experiments (n=2). Source data are provided as a Source Data file.

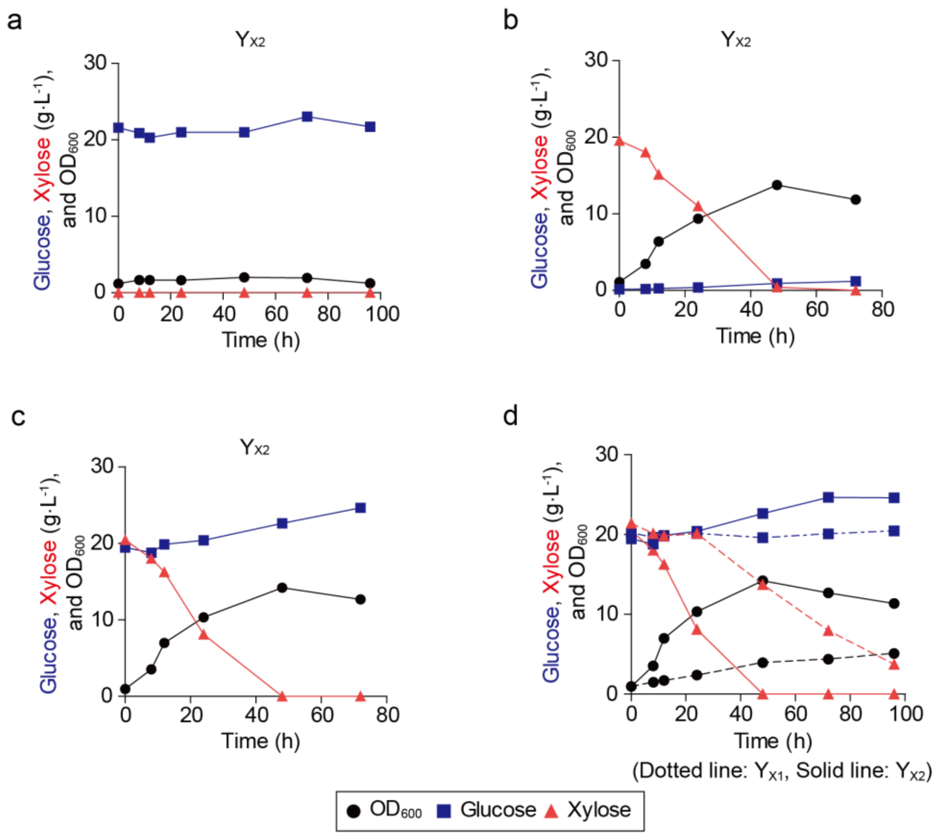

Supplementary Figure 3. Consumption of glucose and xylose by Y_{X2}. Sugar consumption profile of Y_{X2} in (a) YPglucose, (b) YPxylose, and (c) YPglucose+xylose. (d) Sugar consumption profile in YPglucose+xylose of Y_{X1} and Y_{X2} (Dotted line: Y_{X1} and solid line: Y_{X2}). Results are the mean of replicated experiments (n=2). Source data are provided as a Source Data file.

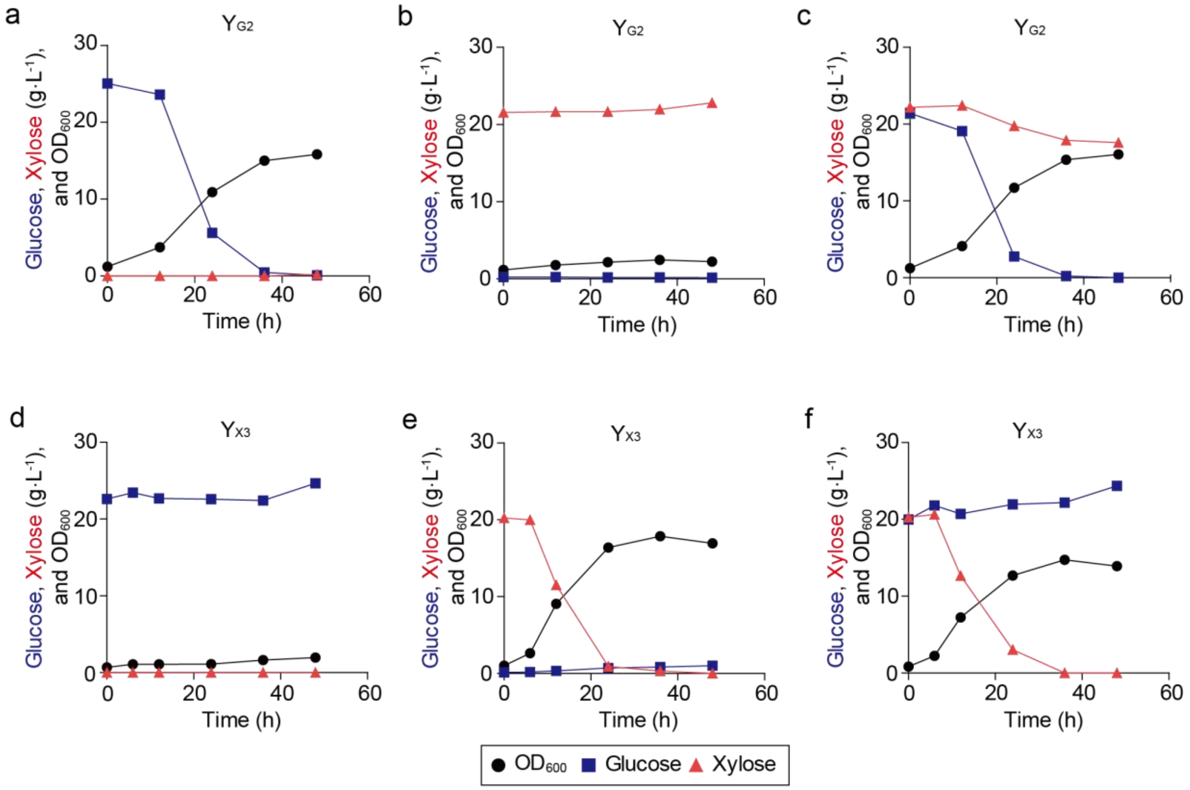

Supplementary Figure 4. Consumption of glucose and xylose by Y_{G2} and Y_{X3}. Sugar consumption profile of Y_{G2} in (a) YPglucose, (b) YPxylose, and (c) YPglucose+xylose. Sugar consumption profile of Y_{X3} in (d) YPglucose, (e) YPxylose, and (f) YPglucose+xylose. Results are the mean of replicated experiments (n=2). Source data are provided as a Source Data file.

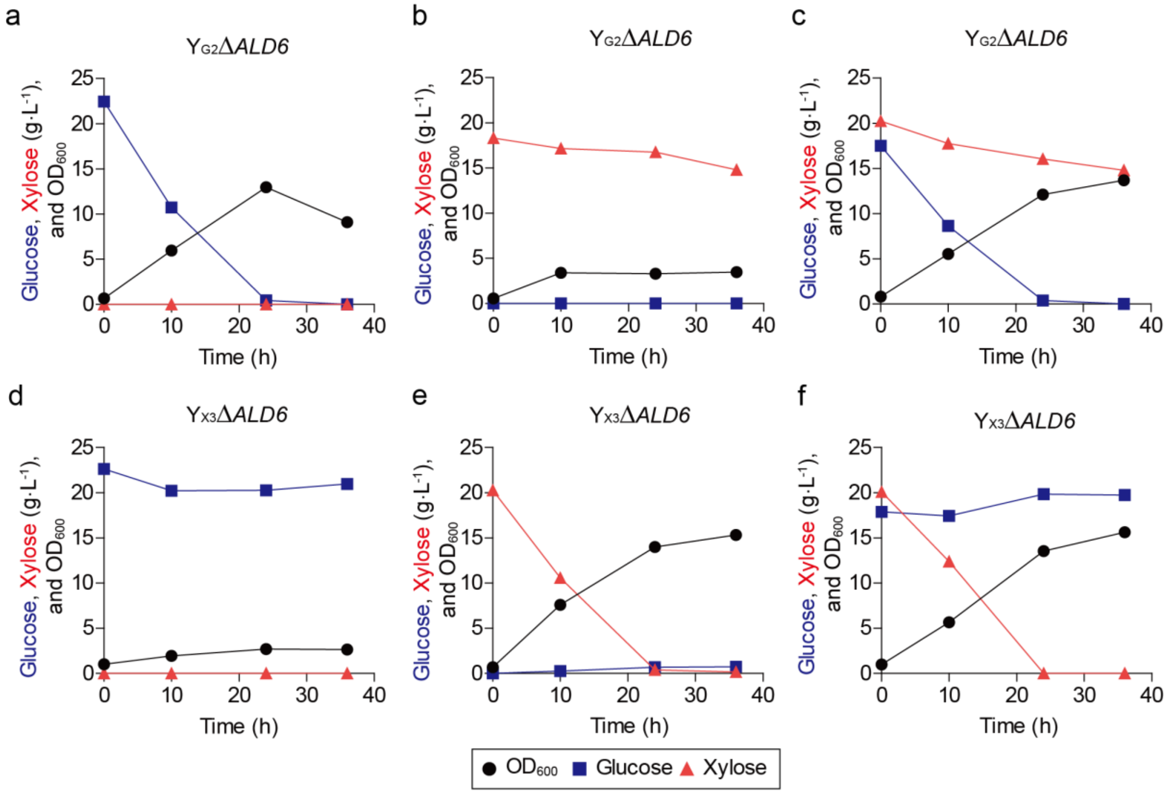

Supplementary Figure 5. Consumption of glucose and xylose by $Y_{G2\Delta ALD6}$ and $Y_{X3\Delta ALD6}$. Sugar consumption profile of $Y_{G2\Delta ALD6}$ in (a) YPglucose, (b) YPxylose, and (c) YPglucose+xylose. Sugar consumption profile of $Y_{X3\Delta ALD6}$ in (d) YPglucose, (e) YPxylose, and (f) YPglucose+xylose. Results are the mean of replicated experiments (n=2). Source data are provided as a Source Data file.

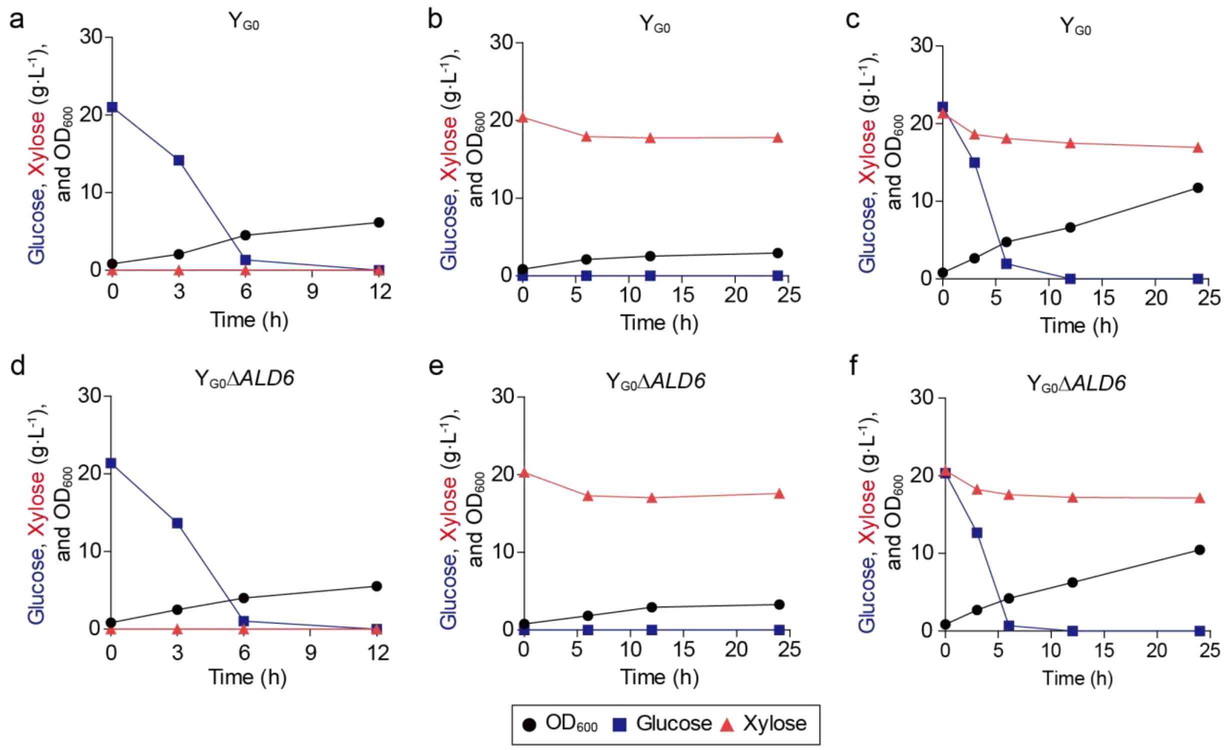

Supplementary Figure 6. Consumption of glucose and xylose in Y_{G0} and $Y_{G0\Delta ALD6}$. Sugar consumption profile of Y_{G0} in (a) YPglucose, (b) YPxylose, and (c) YPglucose+xylose. Sugar consumption profile of $Y_{G0\Delta ALD6}$ in (d) YPglucose, (e) YPxylose, and (f) YPglucose+xylose. Results are the mean of replicated experiments (n=2). Source data are provided as a Source Data file.

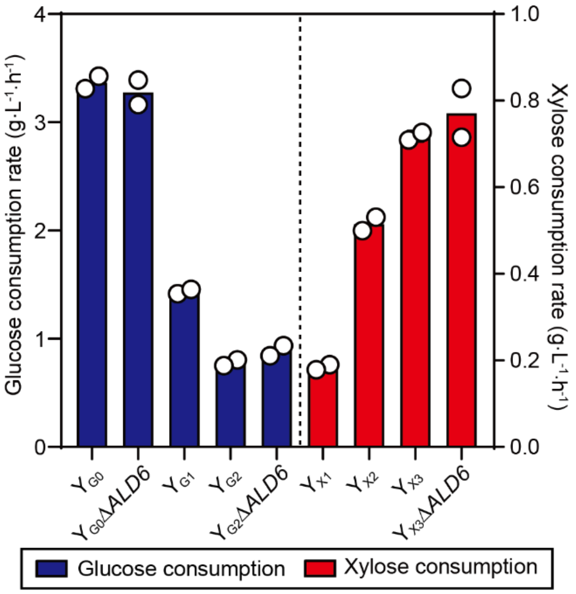

Supplementary Figure 7. The sugar consumption rate of glucose and xylose specialists. All experiments to identify the sugar consumption rate were conducted in YPglucose+xylose and under aeration conditions. Results are the mean of replicated experiments (n=2). Source data are provided as a Source Data file.

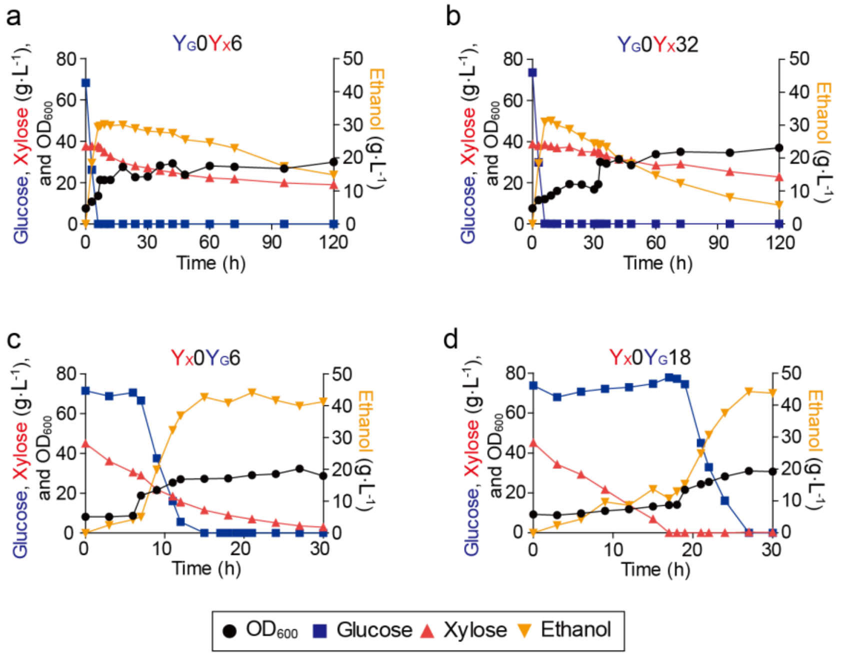

Supplementary Figure 8. Fermentation of glucose and xylose using temporal DOL. (a-b) Fermentation of glucose and xylose with Y_G inoculation at the beginning of fermentation, with subsequent Y_X inoculation at 6 hours (a), 32 hours (b). (c-d) Fermentation of glucose and xylose with Y_X, Y_G inoculation at the beginning of fermentation, with subsequent Y_G inoculation at 6 hours (c), 18 hours (d). $Y_{X3}\Delta$ALD6 was used for Y_X, and $Y_{G0\Delta ALD6}$ was used for Y_G. Results are the mean of replicated experiments (n=2). Source data are provided as a Source Data file.

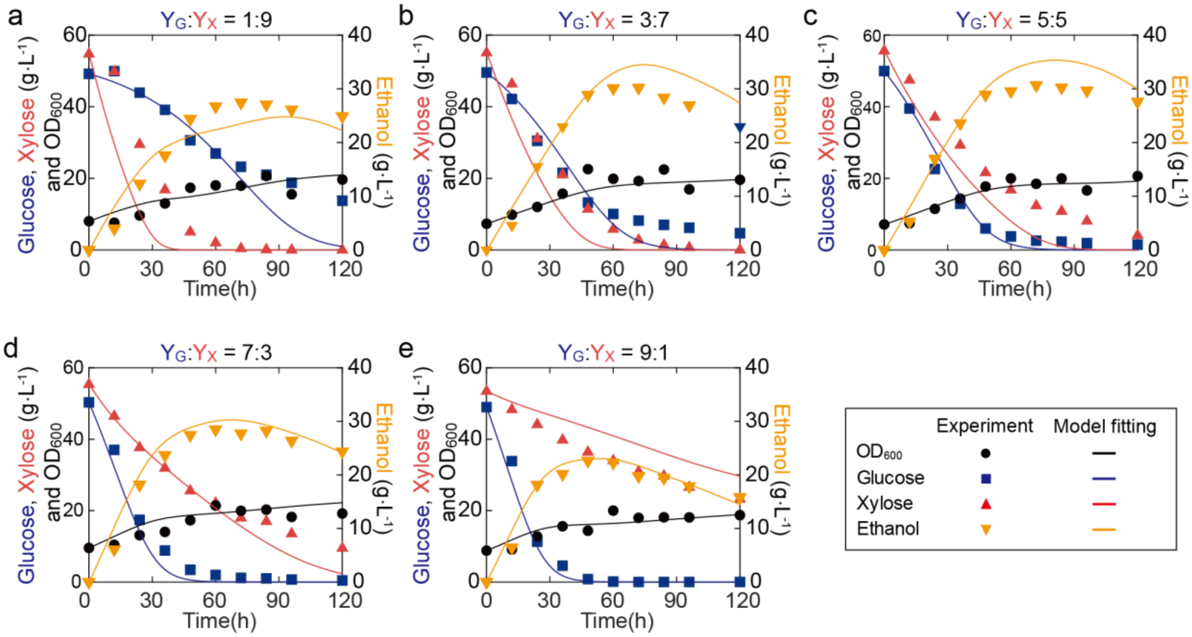

Supplementary Figure 9. Simulated fermentation profiles of the consortium ($Y_{G2\Delta ALD6}$ and $Y_{X3\Delta ALD6}$) with compositional DOL. The initial Y_G:Y_X ratios were 9:1 (a), 7:3 (b), 5:5 (c), 3:7 (d), and 1:9 (e). Colored lines: model fitting; Markers: experimental data. For experimental data, results are the mean of replicated experiments (n=2). Source data are provided as a Source Data file.

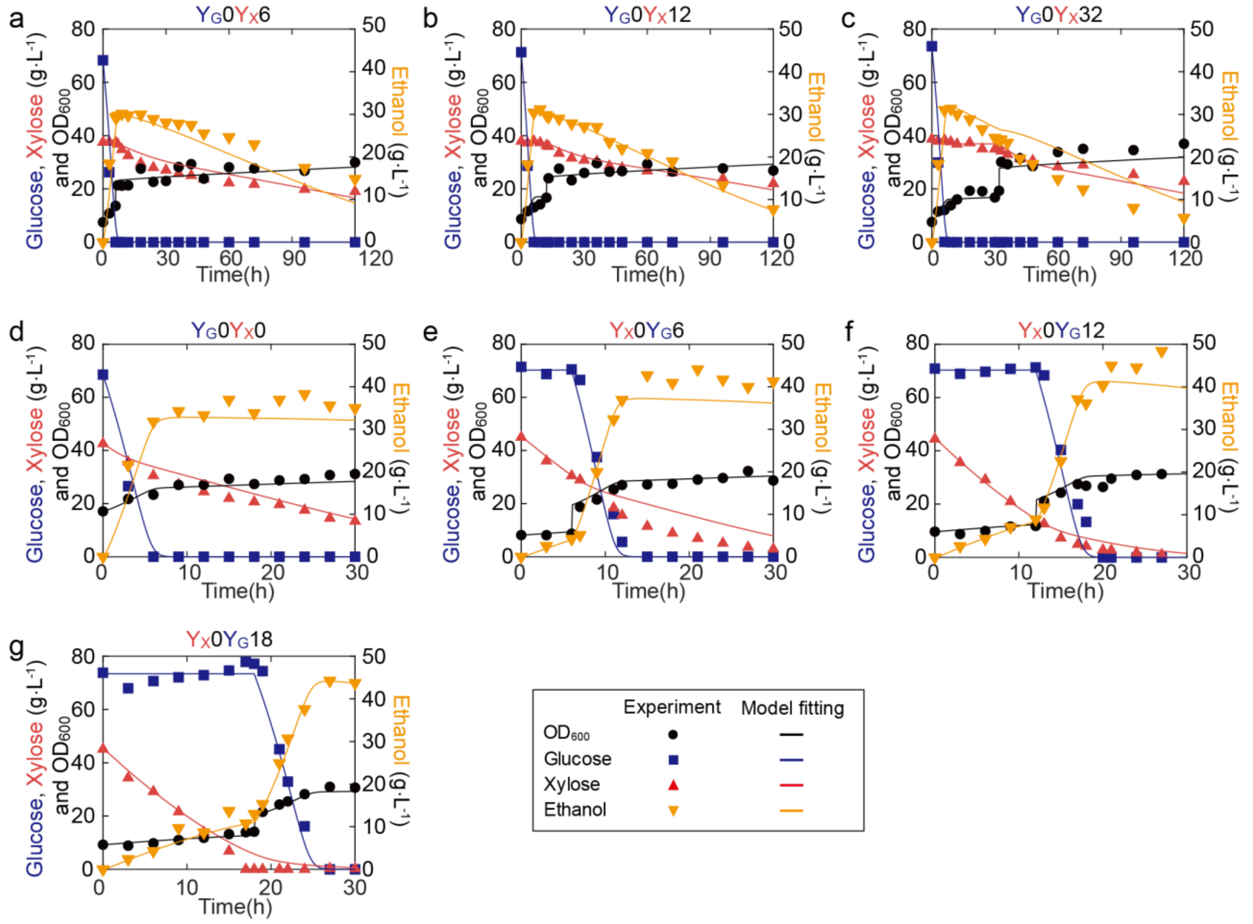

Supplementary Figure 10. Simulated fermentation profiles of the consortium ($Y_{G0\Delta ALD6}$ and $Y_{X3\Delta ALD6}$) with temporal DOL. (a-c) Simulated fermentation profiles when Y_G ($Y_{G0\Delta ALD6}$) was inoculated at the beginning of fermentation while Y_X ($Y_{X3\Delta ALD6}$) was subsequently inoculated at 6 hours (a), 12 hours (b), and 32 hours (c). (d) Simulated fermentation profile when the Y_G and Y_X were inoculated together. (e-g) Simulated fermentation profiles when Y_X was inoculated at the beginning of fermentation while Y_G was subsequently inoculated at 6 hours (e), 12 hours (f), and 18 hours (g). Colored lines: model fitting; Markers: experimental data. For experimental data, results are the mean of replicated experiments (n=2). Source data are provided as a Source Data file.

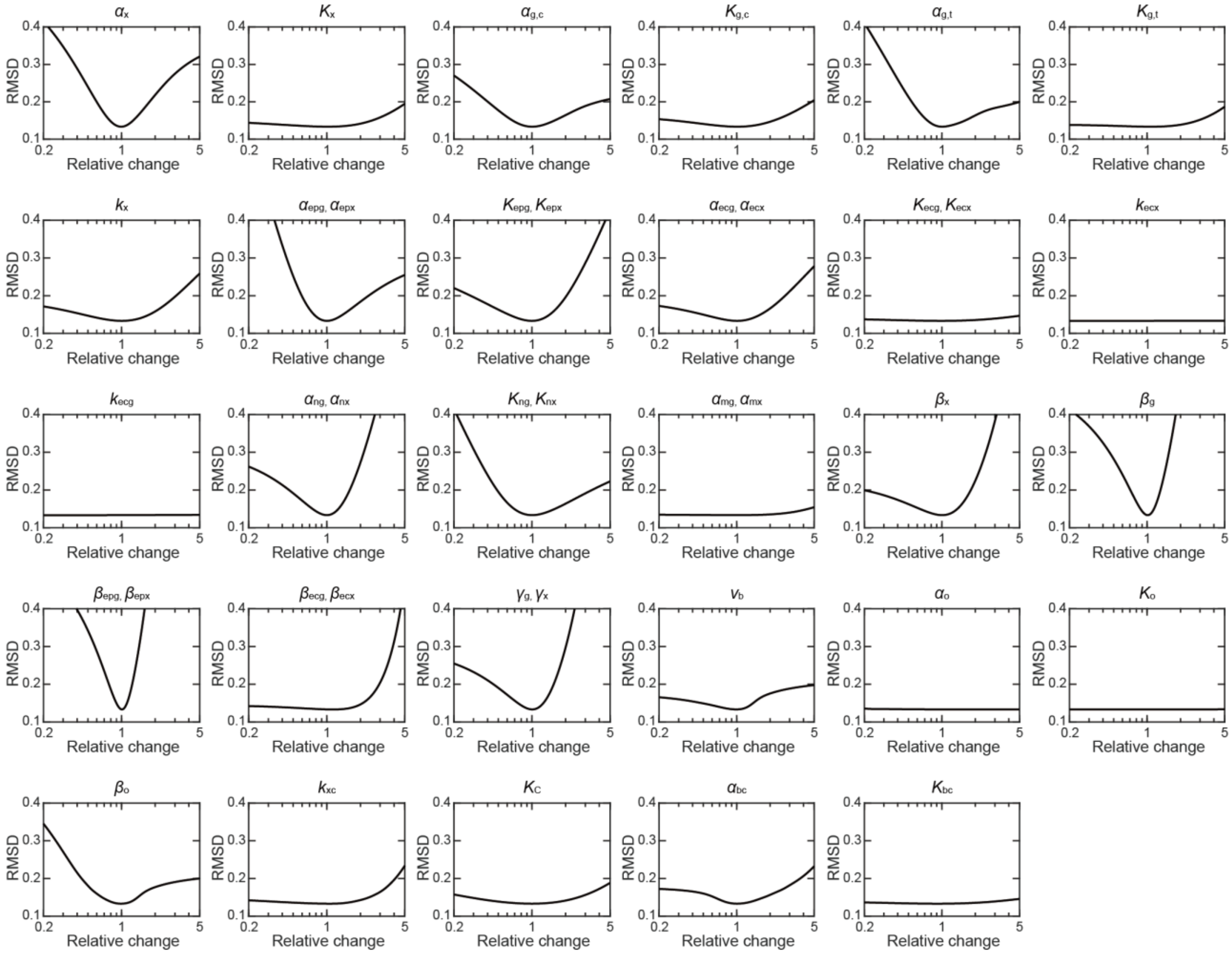

Supplementary Figure 11. Model sensitivity analysis. In each panel, the root-mean-square deviation (RMSD) between model fitting and experimental data is plotted as a function of the relative change of a parameter from 20% to 500% of the inferred value while fixing other parameters at the inferred values. Here, the value of RMSD reflects the model fitting error relative to the mid-range value of the experimental data, the change of RMSD indicates the sensitivity of the model parameters. Source data are provided as a Source Data file.

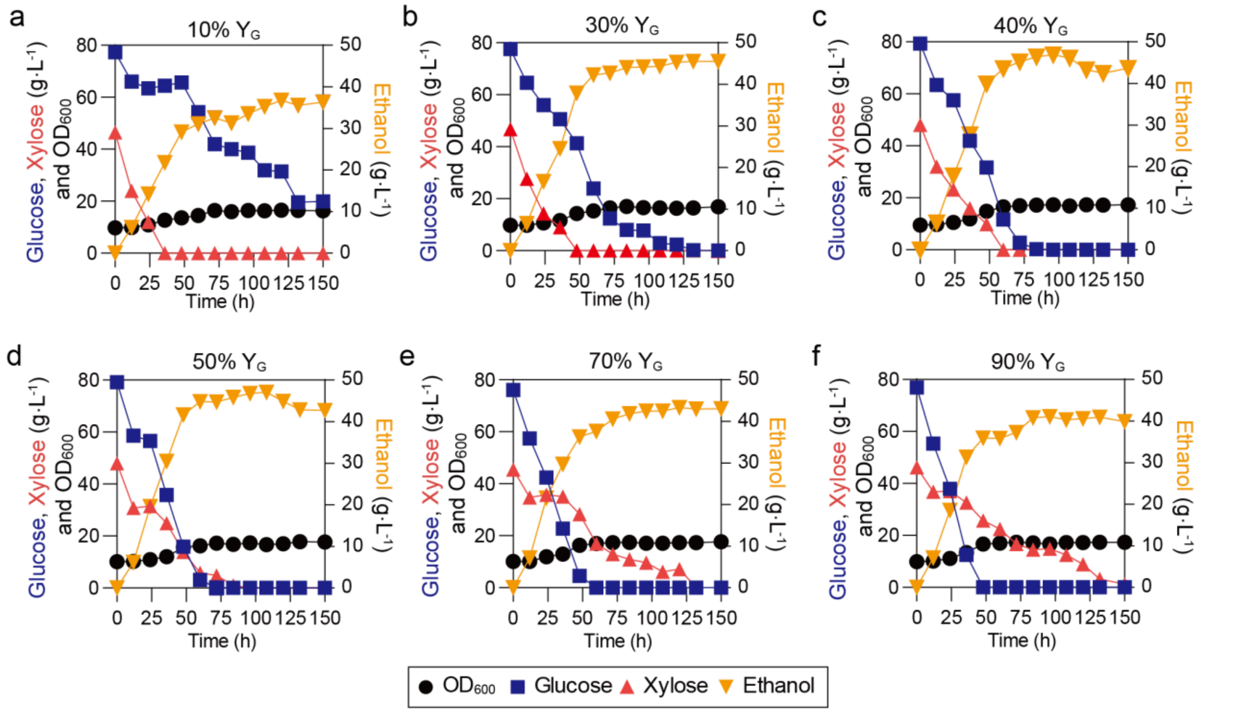

Supplementary Figure 12. Additional compositional DOL fermentations for model validation. The experimental profiles of mixed sugar fermentation with Y_X ($Y_{X3\Delta ALD6}$) and Y_G ($Y_{G2\Delta ALD6}$) with different cell to cell ratio. The initial Y_G:Y_X ratios were 1:9 (a), 3:7 (b), 4:6 (c), 5:5 (d), 7:3 (e), and 9:1 (f). Results are the mean of replicated experiments (n=2). Source data are provided as a Source Data file.

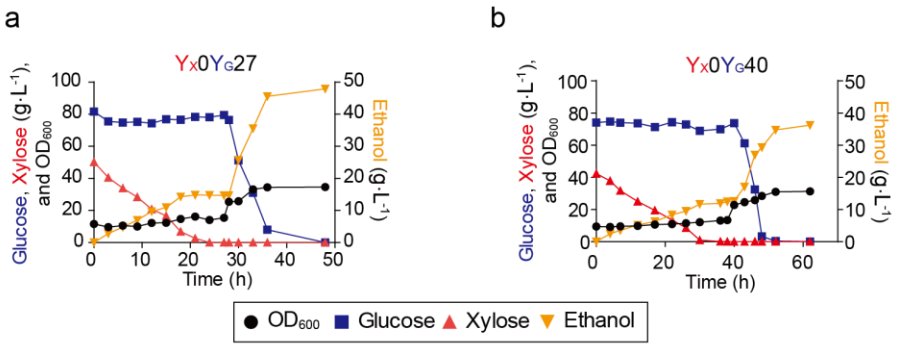

Supplementary Figure 13. Additional temporal DOL fermentations for model validation. In the experimental mixed sugar fermentations, Y_X ($Y_{X3\Delta ALD6}$) was inoculated at the beginning of fermentation while Y_G ($Y_{G0\Delta ALD6}$) was inoculated at 27 hours (a) and 40 hours (b). Results are the mean of replicated experiments (n=2). Source data are provided as a Source Data file.

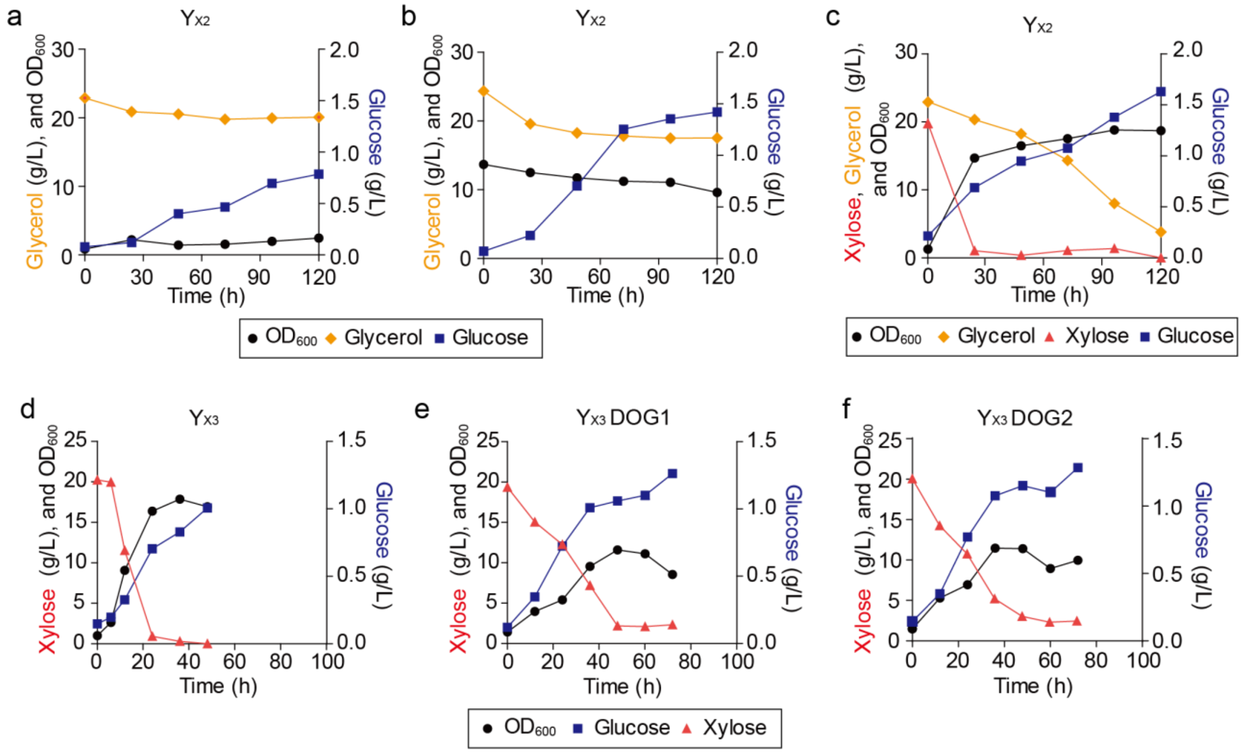

Supplementary Figure 14. Glucose production by a xylose specialist. Sugar consumption and glucose production profile of Y_{X2} under YPglycerol with an initial cell concentration of (a) OD1 and (b) OD10. (c) Sugar consumption and glucose production profile by Y_{X2} under YPxylose+glycerol. Xylose consumption and glucose production of (d) Y_{X3} and (e) Y_{X3}*DOG1*, and (f) Y_{X3}*DOG2*. Results are the mean of replicated experiments (n=2). Source data are provided as a Source Data file.

Supplementary references

1. Eiteman MA, Lee SA, Altman E. A co-fermentation strategy to consume sugar mixtures effectively. *J Biol Eng* **2**, 3 (2008).

2. Chen Y, Wu Y, Zhu B, Zhang G, Wei N. Co-fermentation of cellobiose and xylose by mixed culture of recombinant Saccharomyces cerevisiae and kinetic modeling. *PLoS One* **13**, e0199104 (2018).

3. Verhoeven MD, de Valk SC, Daran JMG, van Maris AJA, Pronk JT. Fermentation of glucose-xylose-arabinose mixtures by a synthetic consortium of single-sugar-fermenting Saccharomyces cerevisiae strains. *Fems Yeast Research* **18**, foy075 (2018).

4. Kim SR*, et al.* Rational and evolutionary engineering approaches uncover a small set of genetic changes efficient for rapid xylose fermentation in *Saccharomyces cerevisiae*. *PLoS One* **8**, (2013).

5. Xu H. Engineering *Saccharomyces cerevisiae* for cellulosic ethanol production. University of Illinois at Urbana-Champaign (2015).

6. Mumberg D, Muller R, Funk M. Yeast vectors for the controlled expression of heterologous proteins in different genetic backgrounds. *Gene* **156**, 119-122 (1995).

7. Kuanyshev N*, et al.* Identification and analysis of sugar transporters capable of co-transporting glucose and xylose simultaneously. *Biotechnol J*, e2100238 (2021).

8. Zhang GC*, et al.* Optimization of an acetate reduction pathway for producing cellulosic ethanol by engineered yeast. *Biotechnol Bioeng* **113**, 2587-2596 (2016).

9. Kwak S*, et al.* Enhanced isoprenoid production from xylose by engineered *Saccharomyces cerevisiae*. *Biotechnol Bioeng* **114**, 2581-2591 (2017).
