# Supplementary material for: Compositional and temporal division of labor modulates mixed sugar fermentation by an engineered yeast consortium
Source: Nat Commun. 2024 Jan 26;15:781. doi: 10.1038/s41467-024-45011-w (PMC10817915; doi:10.1038/s41467-024-45011-w)
Supplement: Supplementary file 2 — Description of Additional Supplementary Files [file 41467_2024_45011_MOESM2_ESM.pdf]

Description of Additional Supplementary Files

File Name: Supplementary Data 1
Description: Variables and parameters of the mathematical model.
